# Atlas Florae Europaeae notes, 33. Taxonomic synopsis of East European species of the *Cytisusratisbonensis* group (Fabaceae)

**DOI:** 10.3897/phytokeys.238.118031

**Published:** 2024-02-23

**Authors:** Alexander N. Sennikov, Valery N. Tikhomirov

**Affiliations:** 1 Botanical Museum, Finnish Museum of Natural History, University of Helsinki, Helsinki 00014, Finland University of Helsinki Helsinki Finland; 2 Belarusian State University, Minsk, Belarus Belarusian State University Minsk Belarus

**Keywords:** Belarus, *Chamaecytisus*, chromosome counts, Leguminosae, mapping, nomenclature, Russia, typification, Ukraine

## Abstract

A group of species of Cytisussect.Tubocytisus with strictly lateral inflorescences, commonly referred to as *C.ratisbonensis* s.l., is critically revised in Eastern Europe on the basis of morphology and comprehensive treatment of herbarium specimens and observations. Seven species and two presumed hybrids are recognised. Complete accounts are provided for each species, with synonyms, typifications, brief morphological descriptions, data on ecology and distributions, taxonomic and nomenclatural annotations. *Cytisuspolonicus* is described as new to science, separated from *C.ratisbonensis* on the basis of morphology and diploid (vs. tetraploid) chromosome count. The lectotype of *C.elongatus* is superseded and a new lectotype is designated; this name has priority for the species previously known as *C.triflorus*. Six species names are newly placed to the synonymy: *Chamaecytisuspineticola* under *Cytisusruthenicus* s. str., and *Cytisusczerniaevii*, *C.leucotrichus*, *C.lindemannii*, *C.ponomarjovii* and *Chamaecytisuskorabensis* under *Cytisuselongatus*. The presumed hybrid between *C.ruthenicus* and *C.elongatus*, which was incorrectly known as *C.czerniaevii*, is described here as *C.semerenkoanus*. *Cytisuslithuanicus*, which has been an obscure name since its original publication, is resurrected for a newly-recognised octoploid species, which is endemic to eastern Poland, western Belarus and north-western Ukraine. The name *C.cinereus* is re-instated for the species previously known as *C.paczoskii*, and *C.horniflorus* is added to its synonymy; its complete distribution area is circumscribed, and its occurrence in Austria, Poland, Romania, Serbia and Slovakia is documented. *Cytisuskreczetoviczii* and *C.elongatus* are reported for the first time from Belarus, and the latter species also from Bosnia and Herzegovina, Montenegro and Slovenia. *Cytisusborysthenicus* and *C.elongatus* are reported as new to some territories in European Russia. *Cytisusratisbonensis* s. str. is treated as absent from Eastern Europe. The neglected protologue of *C.ruthenicus* is discovered, and the nomenclature of all other names is verified and corrected when necessary. The original material of *C.borysthenicus* is re-discovered. Five further lectotypes and one neotype are designated. Distribution areas are circumscribed on the basis of numerous herbarium collections and documented observations, identified or verified by the authors. Chromosome counts published for nameless taxa from Belarus, Ukraine and Russia are assigned to the species according to their herbarium vouchers: *C.borysthenicus*, *C.kreczetoviczii* and *C.lithuanicus* are octoploid (2n = 100), *C.ruthenicus* is tetraploid (2n = 50) and octoploid (2n = 100), and *C.semerenkoanus* and *C.elongatus* are tetraploid (2n = 50).

## Introduction

A group of *Cytisus* (Cytiseae, Fabaceae) with a tubular calyx (C.sect.Tubocytisus DC., *Chamaecytisus* Link) was often treated as a separate genus (Klásková 1958; Holubová-Klásková 1964; Tzvelev 1987). There is no up-to-date phylogeny of Cytiseae Bercht. & J.Presl, a large taxon with uncertain generic limits which underwent a number of major changes in history. The only phylogenetic analysis available (Cubas et al. 2002), which was based on two markers of nrDNA (ITS) and cpDNA (*trn*L-*trn*F) and used rather limited sampling, suggested the integrity of the *Cytisus* group, which can be consequently treated as a single genus. A similar conclusion was reached on the basis of morphology of *Cytisus* s.l. as a whole (Cristofolini 1991; Cristofolini and Conte 2002).

The taxonomic concept in Cytisussect.Tubocytisus had changed dramatically with time. In Eastern Europe, only one or very few species with strictly lateral inflorescences were recognised in the 19^th^ century. Ledebour (1843) and Schmalhausen (1895) accepted a single species only, named *C.biflorus* L’Her., and this concept had been dominant for a long time. Any attempt to separate local taxa (e.g. Gruner (1869a, 1869b); Wołoszczak (1886)) attracted very few followers only (e.g. Paczoski (1914)).

Kreczetowicz (1940) was the first to critically revise the variability and taxonomy of C.sect.Tubocytisus in Eastern Europe (also taking into account the material from Central Europe, Western Siberia and the Caucasus). He noted the diagnostic value of plant habit, flower size and, first of all, pubescence of all parts of the plants. He accepted nearly all previously-described species, added some new taxa and formally named interspecific hybrids. Besides, he introduced the type concept to the group. This revision was promoted by broad-scale taxonomic treatments in “Flora of the USSR” (Kreczetowicz 1941), “Flora of the Caucasus” (Grossheim 1952) and “Flora Europaea” (Heywood and Frodin 1968), which were widely followed in regional treatments. The concept, shaped by Kreczetowicz (1940), became standard in all further revisions including most recent reference books and compilations (Wissjulina 1954; Borisova 1964; Heydemann 1986; Tzvelev 1987; Czerepanov 1995; Nikiforova 2012; Fedoronchuk 2019; Ivanov 2019).

Outside Eastern Europe, these species were treated in some critical revisions. In Poland, Zieliński (1975) accepted a single species with two subspecies, based on the characters of habit. Uncritically following Alexeev (1968), he disregarded the diagnostic value of pubescence and flower size. Skalická (1983) made cursory notes on East European species, but her treatment was based on very few specimens and therefore she was not able to estimate the variability and diagnostic value of the characters. Cristofolini (1991) attempted to make a broad-scale, comprehensive revision of C.sect.Tubocytisus with new infrasectional arrangements and synonymisations. His revision was based on extremely scarce sampling of East European collections, with very few type specimens seen; this fact explains some unconvincing decisions made in this work, which were not accepted by later authors.

Despite recent attempts of further taxonomic splitting (e.g. Ivchenko and Shevera (1992); Ivanov (2019)), practical identification of narrowly-delimited species in this group is very difficult. If not revised by monographers, herbarium collections are often misidentified. The differences in pubescence may be imprecisely described and difficult to apply; these practical difficulties led to an opposition to the approach advocated by Kreczetowicz (1940) and Tzvelev (1987). Alexeev (1968), Yakovlev and Svyazeva (1984), Majorov (2014) and Sagalaev (2018) treated all East European taxa of this group as a single variable species and explained its variability by adaptations to diverse local conditions and clinal variation.

Kreczetowicz (1940) and Tzvelev (1987) noted that hybrids (morphologically intermediate individuals of presumably hybrid origin) occur within a zone where the distribution areas of their presumed parental species overlap. One of such presumed hybrids falls into the variability of polymorphic taxa (i.e. *C.ssyreiszczikovii* V.I.Krecz. and its presumed parent *C.zingeri* (Nenukow) V.I.Krecz. were synonymised with *C.ruthenicus* Fisch. ex Otto: Cristofolini (1991); Sennikov et al. (2021)), whereas three others (*C.kreczetoviczii* Wissjul. interpreted as an intermediate between *C.wulffii* V.I.Krecz. and *C.ruthenicus*: Tzvelev (1987); *C.czerniaevii* V.I.Krecz. = *C.ruthenicus* and *C.lindemannii* V.I.Krecz.: Kreczetowicz (1940); unnamed hybrids between *C.borysthenicus* Gruner and *C.ruthenicus*) are evaluated in the present work.

These taxonomic contradictions and a certain disorder in herbarium collections obscured the taxonomy and distribution of East European species of Cytisussect.Tubocytisus with lateral inflorescences, which, according to different sources, may be known as *C.ratisbonensis* Schaeff., *C.hirsutus* L., *C.ruthenicus* or a number of narrowly and variously defined species. In connection with mapping of this group for Atlas Florae Europaeae, we decided to revise the taxonomy, nomenclature and distributions of its taxa, based on our exhaustive examination of major herbarium collections and literature.

In this particular paper, we examined the taxonomic limits and the species composition of the *C.ratisbonensis* group, which is generally characterised by appressed to subpatent hairs which are densely covering calyces, pedicels, petioles and young branches, and the flowers collected in long racemes of abbreviated axillary fascicles. These characters are widely accepted in the main taxonomic literature (Kreczetowicz 1940; Skalická 1983; Tzvelev 1987; Cristofolini 1991), although may be doubted by some researchers (Yakovlev and Svyazeva 1984; Pifkó and Barina 2016). The taxa previously referred to this group, but excluded in our work, are considered elsewhere (Sennikov and Tikhomirov 2024b).

## Materials and methods

This taxonomic revision used a traditional, morphology-based approach. Diagnostic characters were re-evaluated taking into account the variability observed in herbarium specimens. Taxonomic entities with stable diagnostic characters and certain distribution areas were recognised at species rank, whereas their morphologically intermediate forms found in and around the zone of co-occurrence were treated as presumably hybridogeneous species. Morphological descriptions were compiled on the basis of herbarium specimens and literature. An original identification key and a comparative table were constructed on the basis of these characters.

Distributional areas were revised on the basis of available herbarium collections and documented observations, and taxonomic treatments and checklists were critically evaluated in order to avoid conflicting identifications. Accepted and rejected country-level records are listed in the text under species distribution data; administrative territories or regions are detailed for larger countries. Europe is defined as in Atlas Florae Europaeae (e.g. Kurtto et al. (2018)). Crimea is treated as a separate territory for the purpose of our mapping (as in Kurtto et al. (2018)). Data were collected for complete distribution areas, also outside Eastern Europe.

Herbarium specimens were revised *de visu* or as scanned images via JSTOR (https://www.jstor.org), JACQ Virtual Herbaria (https://www.jacq.org), Muséum national d’Histoire naturelle (https://science.mnhn.fr) and Hungaricana (https://gallery.hungaricana.hu/en/Herbarium); these data were complemented with observations documented by photographs which were available online via iNaturalist (https://www.inaturalist.org/). A complete description of the resulting dataset (3699 specimens or observations) with point distribution maps is published elsewhere (Sennikov and Tikhomirov 2024a). The list of specimens or observations examined (with vouchers documenting our new records) is made available through Internet Archive (Tikhomirov and Sennikov 2023).

All available literature were consulted for nomenclatural novelties and distributional records relevant to *Cytisus* in Eastern Europe. Protologues were analysed, original material and type designations were assessed according to the nomenclatural Code (Turland et al. 2018). Lectotypes or neotypes were designated when no typification had been traced; specimens agreeing in morphological characters with the original descriptions and matching the provenance indicated in the protologues were chosen. Nomenclatural synonyms were cited selectively; more complete lists of homotypic synonyms can be found in Pifkó (2015). Images of most important type collections or representative herbarium specimens are reproduced for each accepted species. The diagnostic characters of the pubescence of each species were illustrated by images from scanned specimens.

As an important biological character supporting the species delimitations, chromosome counts available from Eastern Europe were examined on the basis of published literature (Parfionaŭ et al. 1975; Semerenko 1984). Their herbarium vouchers were traced from MSK and matched against the current taxonomy.

## Results

### Diagnostic characters

The diagnostic characters were extensively discussed by Kreczetowicz (1940) and Cristofolini (1991), and the life forms were studied in detail by Semerenko (2009). We provide our own notes, based on a large set of specimens examined and on field observations. The main diagnostic characters are summarised in Table 1.

**Table 1.** Main diagnostic characters in the *Cytisusratisbonensis* group.

Species	Stems	Branching pattern	Leaflets, shape	Leaflets, pubescence above	Calyx, length (mm)	Calyx, pubescence
*Cytisusborysthenicus*	erect, up to 120(200) cm tall	basal	lanceolate	densely and evenly hairy	10–12	appressed, 0.4–0.6 mm
*Cytisuscinereus*	erect, basally ascending, up to 40–60(80) cm tall	basal	elliptic to obovate	glabrous	11–14	laxly appressed to subpatent, 0.6–1.2(1.5) mm
*Cytisuskreczetoviczii*	erect, up to 80 cm tall	basal	lanceolate to elliptic	sparsely hairy	10–12	(laxly) appressed, 0.4–0.6(0.8) mm
*Cytisuslithuanicus*	erect, basally prostrate, up to 40(60) cm tall	diffuse	obovate	glabrous	12–14	laxly appressed, 0.6–0.8 mm
*Cytisuspolonicus*	prostrate, up to 20 cm above ground	basal	obovate to elliptic	glabrous	(7)8–10	(laxly) appressed, 0.6–0.8(1) mm
*Cytisusratisbonensis*	prostrate, up to 20 cm above ground	basal	obovate to elliptic	glabrous	11–14	laxly appressed, 0.8–1.2(1.6) mm
*Cytisusruthenicus*	erect, up to 120(200) cm tall	basal	obovate	glabrous	10–12	appressed, 0.4–0.6 mm (or absent)
*Cytisussemerenkoanus*	erect, basally ascending, up to 60(80) cm tall	basal	elliptic to obovate	sparsely hairy to subglabrous	10–12	appressed and subpatent, 0.4–0.9 mm
*Cytisuselongatus*	erect, basally ascending, up to 40–60(80) cm tall	basal	elliptic to obovate	densely hairy	11–12	subpatent, 0.8–1.2 mm
*Cytisuswulffii*	prostrate, up to 20 cm above ground	diffuse	obovate to oblong	hairy	14–15	laxly appressed, 0.5–1 mm

#### Life form and habit

All species are shrubs of small or medium size with lignified stems, typically with no main trunk, which differ in growth type and branching pattern of their twigs.

Some species (*C.polonicus* Sennikov & Val.N.Tikhom., *C.ratisbonensis*, *C.wulffii*) have main stems which are predisposed for prostration, thus forming horizontally growing, apically ascending branches. Such prostrate shrubs grow over rocky grounds in mountainous areas.

The other species with generally erect stems can be classified according to the length of ascending basal parts of their main stems, forming compact or lax shrubs. *Cytisusborysthenicus* and *C.ruthenicus* have basally suberect stems and very little tendency to ascending. *Cytisuscinereus* Host and *C.elongatus* Waldst. & Kit. have basally ascending stems that run shortly underground, thus forming lax shrubs. The main stems in *C.lithuanicus* Gilib. are long ascending; when their basal parts are covered by soil, they may produce adventitious nodal roots, with a large part of the shrub thus being underground; this type of shrub is transitional to prostrate.

The branching pattern of stems may be basal (*C.borysthenicus*, *C.cinereus*, *C.elongatus*, *C.polonicus*, *C.ratisbonensis*, *C.ruthenicus*) with rather long and thick branches, or diffuse (*C.lithuanicus*, *C.wulffii*) with shorter and thinner branches.

The plant height differs considerably. The prostrate shrubs (*C.polonicus*, *C.ratisbonensis*, *C.wulffii*) ascend up to 20 cm above the ground. The compact erect shrubs (*C.borysthenicus*, *C.ruthenicus*) may grow very robust, up to 150 cm tall, whereas the lax erect shrubs (*C.cinereus*, *C.elongatus*) are typically lower, up to 60(80) cm tall. The semi-prostrate shrubs (*C.lithuanicus*) are up to 40(60) cm tall.

#### Inflorescence

This revision is limited to the species with a single type of inflorescence, i.e. lateral. Flowers are collected in small axillary fascicles, which are borne on lignified twigs of the previous year; flowering occurs in late summer. As a rule, no flowers are borne on the new growth of twigs. Exceptions are extremely uncommon; we have seen only one specimen of *C.cinereus* that abnormally developed apical inflorescences on the new growth in secondary flowering.

#### Flowers

Flowers are pedicellate, pedicels of various lengths. There is a tendency for certain species to produce longer (*C.lithuanicus*) or shorter (*C.polonicus*) pedicels, but this character is too variable and cannot be reliably used as diagnostic because the pedicel length depends on the flowering period and ecological conditions.

Corolla is of various shades of yellow (Tzvelev 1987), which cannot be reliably observed in dry collections. The length of corolla is variable; some species have noticeably smaller (e.g. *C.polonicus*) or larger (e.g. *C.ratisbonensis*) flowers. The standard may be glabrous or variously pubescent. This may be an auxiliary diagnostic character in some species pairs (*C.ruthenicus* with glabrous standard and *C.borysthenicus* with hairy standard), although this difference is blurred because of the variability in the other species (*C.cinereus*, *C.elongatus*). Size of flowers and type of pubescence are most easily observed in calyces, and we recommend these characters for identification keys.

#### Leaves

Leaves are composite, of three leaflets which are mostly obovate to nearly elliptic in most species, except *C.borysthenicus* in which the leaflets are lanceolate or narrowly lanceolate. The leaflets are invariably glabrous or hairy above, except for presumed hybrids, in which the leaflets can be variously hairy to subglabrous. This character is easy to observe and clearly diagnostic.

#### Pubescence

Pubescence is a key character that distinguishes taxa at the level of species, especially in East European treatments (Kreczetowicz 1940; Tzvelev 1987). It is invariably present in all species, except for *C.ruthenicus*, in which a glabrous morphotype is known and described as C.ruthenicusvar.zingeri Nenukow. Such plants are connected with the hairy morphotype by intermediate forms and, therefore, deserve the rank of variety (Sennikov at al. 2021).

**Figure 1.**
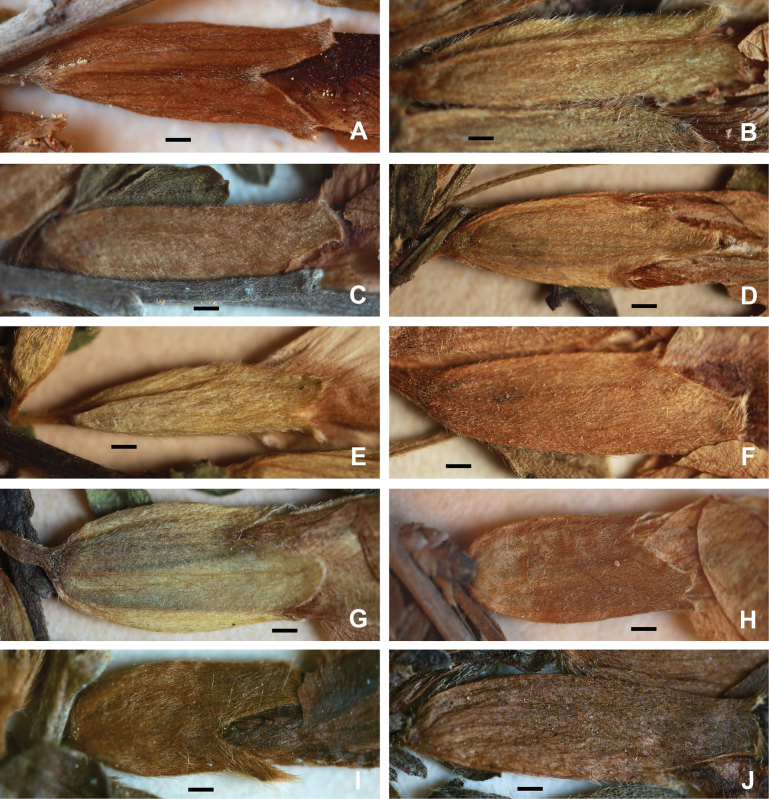
Pubescence on calyces in the *Cytisusratisbonensis* group **A***C.borysthenicus***B***C.cinereus***C***C.kreczetoviczii***D***C.lithuanicus***E***C.polonicus***F***C.ratisbonensis***G***C.ruthenicus***H***C.semerenkoanus***I***C.elongatus***J***C.wulffii*. Scale bars: 1 mm.

The type of pubescence on young branches, pedicels and calyces is most characteristic of certain species (Fig. 1). It may be composed of appressed or subappressed hairs of various lengths; the length of hairs is fixed within a certain range and can be used for species identification. The shortest hairs (0.2–0.4 mm, *C.ruthenicus*) are appressed, whereas longer hairs tend to be spreading and less appressed to subpatent when their length increases (0.6–1.2(1.6) mm, *C.cinereus*). Patent hairs are a distinct type of pubescence which is characteristic of the *C.hirsutus* group; such hairs are erect and very long (1.5–2.2 mm).

### Chromosome counts

There are very few reports on chromosome numbers in Cytisussect.Tubocytisus from Eastern Europe. In those cases when vouchers were traced, this information proved to be informative and taxonomically valuable.

Forissier (1973) reported an octoploid chromosome count for *C.ruthenicus*, based on cultivated material originating from Central Russia (two samples from Moscow and Riazan Regions). This material has not been examined, and its taxonomic identity is doubtful.

Parfionaŭ et al. (1975) made an extensive sampling of C.sect.Tubocytisus in Belarus for chromosome counts because of taxonomic difficulties and uncertain species limits in this group. They counted chromosome numbers in 24 individuals identified as *Chamaecytisus* sp. in Belarus and in two individuals identified as *C.ruthenicus* in Ukraine.

Based on the combination of the chromosome counts and morphology, Semerenko (1984) inferred the existence of different, yet poorly understood taxa in Belarus. She distinguished one widespread tetraploid and two octoploids with limited distributions in the south-western and south-eastern parts of the country.

In the absence of taxonomic expertise, Parfionaŭ et al. (1975) were not able to identify their samples of *Cytisus* to the level of species. Based on the voucher specimens at MSK, we can provide the following identifications: 2n = 50 (Minsk, Gomel, Grodno Regions of Belarus, Zhitomir Region of Ukraine) – *Cytisusruthenicus*, 2n = 50 (Gomel Region) – *C.semerenkoanus*, 2n = 100 (Brest Region) – *C.lithuanicus*, 2n = 100 (Gomel Region) – *C.ruthenicus* and *C.kreczetoviczii*.

Similarly, we decipher the following chromosome counts included in Semerenko (1984): 2n = 100 (Ukraine) – *C.borysthenicus*, 2n = 50 (Kursk and Lipetsk Regions of Russia) – *C.elongatus*.

### Putative hybridisation

Hybridisation and polyploid formation were a key factor in evolution of plant taxonomic diversity (Soltis and Soltis 2009). High polyploid chromosome numbers in Cytisussect.Tubocytisus suggest that hybridisation may have played an important role in speciation of this group. At present, in spite of rather inconspicuous morphological differences, most of its species are clearly delimited. Morphologically intermediate individuals of presumably recent hybrid origin are observed between *C.borysthenicus* and *C.ruthenicus* (*C.kreczetoviczii*) and between *C.ruthenicus* and *C.elongatus* (*C.semerenkoanus*). Such individuals are found co-occurring in mixed populations of the parental taxa, but also without connection to the presumed parents.

Herbarium specimens of *C.kreczetoviczii* are observed to have lower seed set, which may indicate partial hybrid sterility. However, no experimental studies have been performed to prove this observation.

### Taxonomy and nomenclature

#### 1. Cytisus ruthenicus

Taxon classificationPlantaeFabalesFabaceae

Fisch. ex Otto in Allg. Gartenzeit. 12: 347 (1844)

7908602F-3596-5107-B180-0C835C994C9B

– Cytisus ratisbonensis subsp. ruthenicus (Fisch. ex Otto) Syr. in Trudy Bot. Sada Imp. Yur’evsk. Univ. 13(1–2): 209 (1912) – Chamaecytisusruthenicus (Fisch. ex Otto) Klásk. in Preslia 30: 214 (1958) – Chamaecytisusratisbonensissubsp.ruthenicus (Fisch. ex Otto) Ziel. in Arbor. Kórnickie 20: 78 (1975).
= Cytisus ruthenicus var. zingeri Nenukow in Litvinov, Spisok Rast. Gerb. Russk. Fl. Bot. Muz. Rossiisk. Akad. Nauk 8(52): 1 (1916) – Cytisuszingeri (Nenukow) V.I.Krecz. in Bot. Zhurn. SSSR 25: 260 (1940) – Chamaecytisuszingeri (Nenukow) Klásk. in Preslia 30: 214 (1958). Type. Russia. Nizhni Novgorod Region, Balakhna District. Chernoretsk State Forest District, pine forests on sands, 22.06.1914, *I.M. Shvetsov* [Herbarium Florae Rossicae No. 2552(pt.)] (lectotype LE01024070, two fragments from the right (with well-developed leaves and pods), designated by Sennikov and Tikhomirov in Sennikov et al. (2021: 58); isolectotypes H1279755, KW000114831, KW000114832, LE01024071, LE01024072, M0210776, MW0593001, NNSU, NS0031789, and many other collections).
= Cytisus ssyreiszczikovii V.I.Krecz. in Bot. Zhurn. SSSR 25: 261 (1940) – Chamaecytisusruthenicusvar.ssyreiszczikovii (V.I.Krecz.) Tzvelev, Fl. Evropeiskoi Chasti SSSR 6: 222 (1987) – Chamaecytisusssyreiszczikovii (V.I.Krecz.) Vasjukov & Tatanov in Turczaninowia 19: 67 (2016). Type. Russia. Ulianovsk Region and District. Belyi Klyuch Village, mixed forest with oak on the watershed between Volga and Sviyaga Rivers, 02.08.1917, *A.P. Shennikov* (lectotype LE01017901, designated by Vasjukov and Tatanov (2016: 67)).
= Chamaecytisus pineticola Ivchenko in Ukr. Bot. Zhurn. 49: 84 (1992), syn. nov. Type. Ukraine. “In adjacentibus Kioviae, prope Irpenj, margines pineti,” 25.05.1976, *I.S. Ivchenko* (holotype KW).

##### Type.

**Figure 2.**
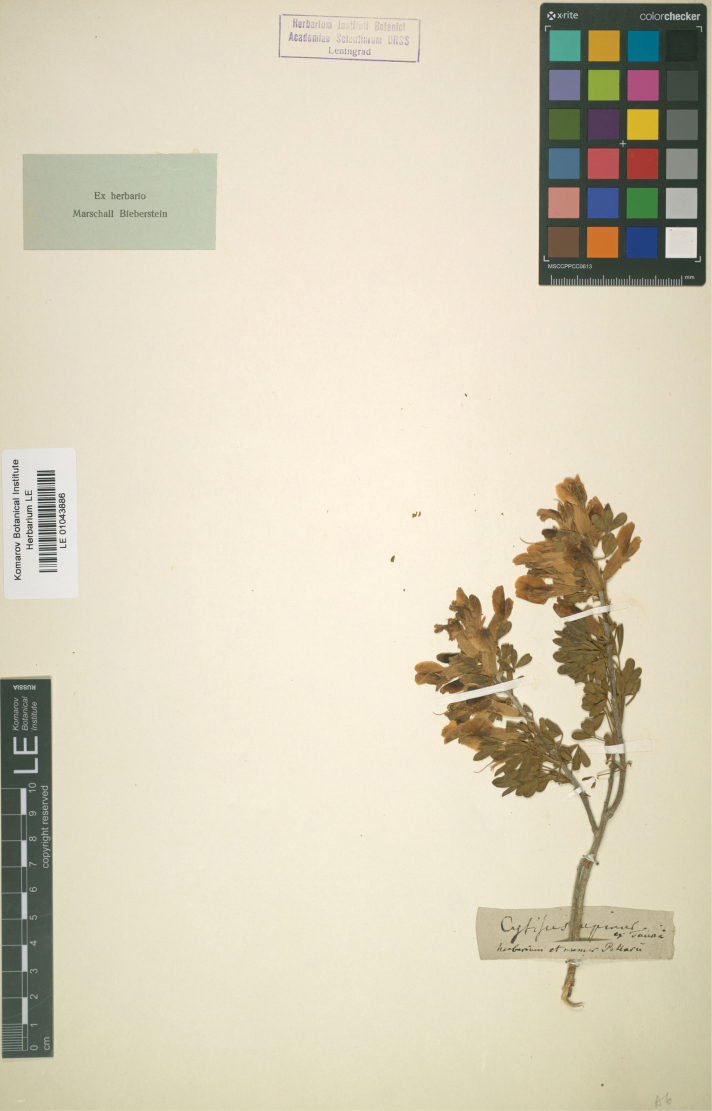
Lectotype of *Cytisusruthenicus* Fisch. ex Otto.

Crimea. “Ex Tauria”, *P.S. Pallas* in Herb. Bieberstein (lectotype LE01043886, designated here). Fig. 2.

##### Description.

Upright shrubs with erect stems up to 120(200) cm tall and long branches. Leaves with obovate leaflets, glabrous above, with appressed hairs 0.2–0.4 mm long below, petioles sparsely covered with appressed hairs. Flowers strictly lateral, 1–4 in axils, on pedicels 5–7 mm long, yellow; calyx 10–12 mm long, with appressed hairs 0.4–0.6 mm long; standard suborbicular, glabrous above.

##### Distribution.

Europe: Poland (Zieliński 1975; Danielewicz 2020), Moldova (Heydemann 1986), Belarus (Semerenko 1999; Dubovik 2016), Ukraine (Tzvelev 1987; Fedoronchuk 2019, 2022), Crimea (Yena 2012), Russia (central, southern and south-eastern parts: Tzvelev 1987), Kazakhstan (north-western part: Tulaganova 1981; Abdulina 1999). Asia: Russia (south-western Siberia: Kurbatsky 1994; northern Caucasus: Zernov 2006), Georgia, Kazakhstan (north-western and northern parts: Tulaganova 1981). Apparently, the species is present also in Slovakia (Holub and Bertová 1988), although the relevant herbarium material has not been revised. Its presence in Hungary and Romania is also expected.

##### Ecology.

In the forest zone, the species is largely confined to rather dry pine and mixed forests, growing mostly in open places (forest margins and clearings); in the forest steppe and steppe zones, the species is found in open places in forested dry creeks.

##### Chromosome counts.

2n = 50 (Parfionaŭ et al. 1975, as *Chamaecytisus* sp. and *C.ruthenicus*); material collected from native populations in Gomel, Grodno and Minsk Regions of Belarus and Zhitomir Region of Ukraine; vouchers at MSK. 2n = 100 (Semerenko 1984); material collected from native populations in Gomel Region; vouchers at MSK. Dubious record: 2n = 100 (Forissier 1973, as *Chamaecytisusruthenicus*); material received from the Main Botanical Garden in Moscow, originating from Moscow and Riazan Regions of Russia; vouchers unknown.

##### Notes on nomenclature.

*Cytisusruthenicus* was originally named by F. von Fischer who cultivated plants from the southern course of the Volga River and the southern Ural Mountains in the private botanical garden of Count Alexei Razumovsky. Fischer cultivated rather variable plants received from various collectors, evidently from Friedrich Helm (the Urals) and possibly from Johan Peter Falk (Volga). As evident from herbarium vouchers, subsequently transferred from Gorenki to the Imperial Botanical Garden in St. Petersburg, Fischer introduced the plants from Volga under the provisional name “*Cytisussupinus* s. *volgensis*” (Fischer 1808: 110, 1812: 68). The epithet “ruthenicus” appeared later on herbarium labels and with seeds distributed by Fischer; for the first time, it appeared in print in the first catalogue of plants cultivated in the Botanical Garden in Petersburg (Fischer 1824: 25). Since then, it was mentioned in a number of publications, all without any descriptive matter.

Wołoszczak (1886) has been commonly cited as the place of valid publication of *C.ruthenicus*, also by those who published new nomenclatural combinations based on this species name. The material used and distributed by Wołoszczak (Kerner 1893) largely belongs to *C.cinereus*, with a minor admixture of *C.ruthenicus*. Nevertheless, the species name was validly published earlier (Otto 1844) with a sole reference to an extensive description under *C.supinus* M.Bieb. non L. (Marschall von Bieberstein 1819), which is referable to the same plants as intended by Fischer.

Under *C.supinus*, Marschall von Bieberstein (1819: 476) described plants with foliose inflorescences and appressed pubescence on calyces and pedicels, and hairy pods. He discussed Fischer’s plants named “*Cytisussupinus* s. *volgensis*” as a variety of his species. In the personal collection of Bieberstein at LE, there is a specimen labelled “*C.supinus*” and collected from “Tauria” (Crimea), which is in complete agreement with the characters stated by Bieberstein and represents a typical specimen of *C.ruthenicus* as currently understood (Tzvelev 1987). This specimen is designated as a lectotype of *C.ruthenicus* here.

In spite of the change in the presumed basionym, all combinations published without references to the actual basionym or explicitly based on *C.ruthenicus* “Fisch. ex Woł.” are validly published as based on *C.ruthenicus* Fisch. ex Otto under Art. 41.4 and 41.8(a).

##### Notes on taxonomy and distribution.

The name *Cytisuszingeri* belongs to a variety with completely glabrous pods, branches and leaves, which is known from several localities at the confluence of Oka and Volga Rivers in Nizhni Novgorod and Vladimir Regions and in two localities in Kurgan Region (Sennikov et al. 2021). This variety has no separate distribution area, commonly co-occurs with the hairy plants at the same locality (Nenukow 1916), and plants with intermediate characters are common.

*Cytisusssyreiszczikovii* was described as a presumed hybrid between *C.ruthenicus* and *C.zingeri*; in our circumscription, such less hairy plants clearly fall within the variability of the species.

*Cytisusruthenicus* was frequently confused with *C.ratisbonensis* because of their leaves glabrous above; it differs from the latter by upright, taller stems and a longer pubescence on young shoots, petioles and calyces. *Cytisusruthenicus* has not been formally reported from Romania, but apparently passed under the misapplied name C.ratisbonensisvar.biflorus in Grinţescu (1957).

Similarly, its presence of Slovakia was implied by Holub and Bertová (1988), who noted the occurrence of taller plants in the eastern part of the country.

In the Caucasus, *C.ruthenicus* was included in *C.caucasicus* (Grossheim 1952; Gvinianidze 1981), which was synonymised with *C.ruthenicus* by Tzvelev (1987). *Cytisuscaucasicus* was described as different from *C.ruthenicus* in a greater pubescence of the plant, which is less appressed and longer than in the latter species (Grossheim and Schischkin 1928). Our revision of herbarium collections confirms a broad distribution of *C.ruthenicus* in the Caucasus and its separation from *C.caucasicus*.

*Chamaecytisuspineticola* was distinguished from *C.ruthenicus* by its occurrence in Ukrainian pine forests rather than Russian steppes and by presumed differences in the density of pubescence and flower size (Ivchenko and Shevera 1992). As evident from the protologue, the authors misapplied the name *C.ruthenicus* to *C.cinereus*, because the collections distributed by Wołoszczak under *C.ruthenicus* belong to *C.cinereus*, and their comparisons are, therefore, incorrect. Besides, the authors compared their new species with *C.borysthenicus*, which was presumably different in a denser pubescence, broader leaflets and nearly glabrous standard. The scattered pubescence on the upper side of its lanceolate leaflets indicated in the protologue (Ivchenko and Shevera 1992) corresponds to the hybrid between *C.borysthenicus* and *C.ruthenicus*, which is quite common along the Dnepr River, but authentic specimens from the type population (KW) undoubtedly belong to *C.ruthenicus* s. str.

#### 2. Cytisus kreczetoviczii

Taxon classificationPlantaeFabalesFabaceae

Wissjul. in Zerov, Fl. URSR 6: 586 (1954)

5D6C039C-7645-5A5F-B7FE-D4285DC651E1

– Chamaecytisus kreczetoviczii (Wissjul.) Holub in Folia Geobot. Phytotax. 11: 83 (1976) – Chamaecytisusruthenicusvar.kreczetoviczii (Wissjul.) Skalická in Rad. Akad. Nauka Um. Bosne Hercegovine 72: 241 (1983) – Cytisusruthenicussubsp.kreczetoviczii (Wissjul.) Cristof. in Webbia 45: 214 (1991).

##### Type.

**Figure 3.**
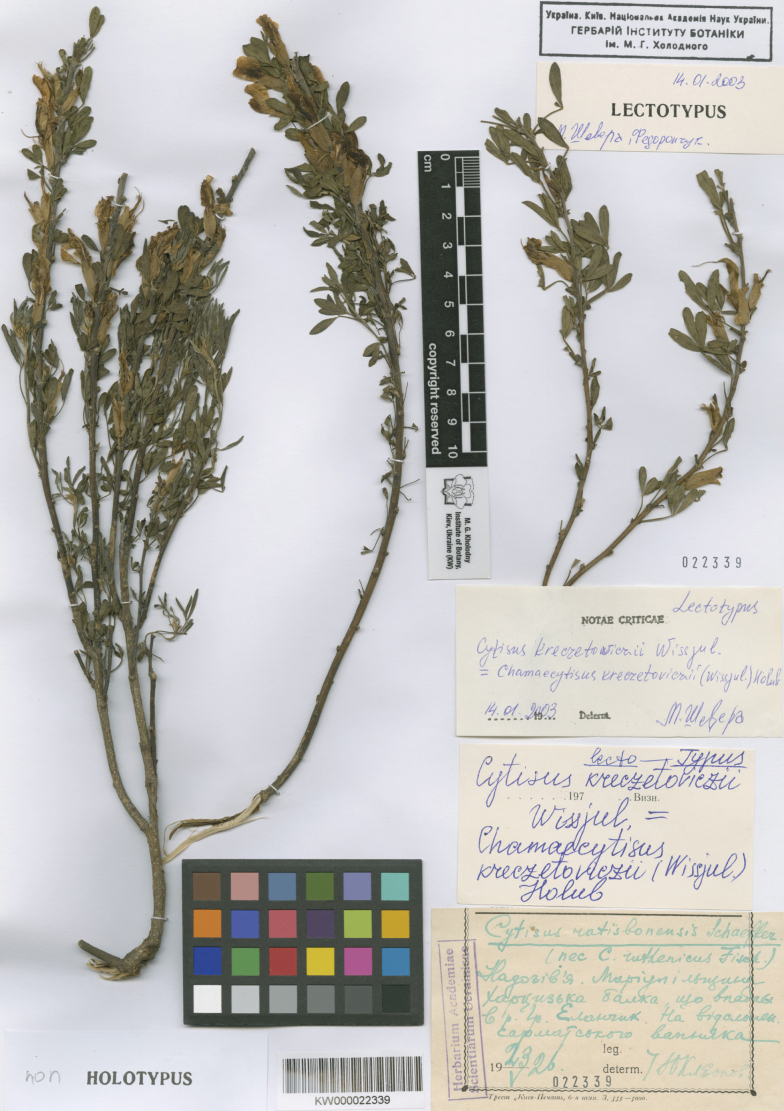
Lectotype of *Cytisuskreczetoviczii* Wissjul.

Ukraine. “Prope flum. Gruzkyj Jelanczyk, loco Charcysska balka dicto, in decliviis calcareis sarmaticis,” 23.05.1926, *Yu.D. Kleopov* (lectotype KW000022339, designated by Krytzka et al. (1999: 610); isolectotypes KW000022338, KW000022340, possible isolectotype KW000022341). Fig. 3.

##### Description.

Upright shrubs with erect stems up to 80 cm tall and long branches. Leaves with lanceolate to elliptic leaflets, sparsely hairy above, with appressed hairs 0.1–0.2(0.4) mm long below, petioles sparsely covered with laxly appressed (partly subpatent) hairs. Flowers strictly lateral, 1–4 in axils, on pedicels 4–6 mm long, yellow; calyx 10–12 mm long, with (laxly) appressed hairs 0.4–0.6(0.8) mm long; standard suborbicular, glabrous or sparsely hairy above.

##### Distribution.

Europe: Belarus (new record), Ukraine, Russia (Tzvelev 1987). Reported for the first time from Belarus here.

##### Ecology.

Alluvial sands in larger river valleys, riverside slopes, often on exposed calcareous substrates.

##### Chromosome counts.

2n = 100 (Parfionaŭ et al. 1975, as *Chamaecytisus* sp.); material collected from native populations in Gomel Region; vouchers at MSK.

##### Notes on nomenclature.

The type specimen of *Cytisuskreczetoviczii* was interpreted as holotype by Krytzka et al. (1999: 610). Since the holotype specimen was not indicated in collections by the author and the type collection was represented by multiple duplicates, Fedoronchuk et al. (2003: 96) formally designated a lectotype. However, in this case, the earlier holotype indication is correctable to lectotypification because of its having been published prior to 2001 (Turland et al. 2018).

##### Notes on taxonomy and distribution.

This is a variable taxon, which occupies an intermediate position between *C.borysthenicus* and *C.ruthenicus* in the shape of leaves and the pubescence of the upper side of leaves. Taxonomically, these plants were recognised as a locally endemic species in Ukraine (Wissjulina 1954) and as an unnamed hybrid in Russia (Tzvelev 1987). Tzvelev (1987) misinterpreted *C.kreczetoviczii* as another alleged hybrid, between *C.ruthenicus* and *C.wulffii*. The latter taxon has hairy upper surfaces of leaves, but its creeping habit and a narrowly restricted distribution in the mountainous Crimea makes its participation in any hybridisation outside the mountains highly unlikely. *Cytisuskreczetoviczii* has tall and erect branches and narrowly lanceolate leaves (Wissjulina 1954), and its occurrence within the overlapping distributions of *C.ruthenicus* and *C.borysthenicus* agrees with its intermediate morphology between the two latter species. The reduction of this taxon to *C.ruthenicus*, as proposed by Skalická (1983) and Cristofolini (1991), is not justified because *C.kreczetoviczii* differs from *C.ruthenicus* by its stems, petioles and pedicels covered with subappressed hairs 0.6–0.8 mm long (vs. 0.4–0.6 mm long in *C.ruthenicus*) and its lanceolate to elliptic (vs. obovate) leaflets variously hairy (vs. glabrous) above. This taxon largely occurs in mixed populations together with its parental species, although some localities (including the type one) can be found without direct connection with the parents. It advances further northwards than *C.borysthenicus* and occurs in Belarus in the absence of the latter.

#### 3. Cytisus borysthenicus

Taxon classificationPlantaeFabalesFabaceae

Gruner in Bull. Soc. Imp. Naturalistes Moscou 41(4): 446 (1869)

314E3AE1-3EDF-547B-99FD-DCDD9C9E006C

– Cytisus biflorus subsp. borysthenicus (Gruner) Pacz. in Trudy Bot. Sada Imp. Yur’evsk. Univ. 15(2–3): 95 (1914) – Chamaecytisusborysthenicus (Gruner) Klásk. in Preslia 30: 214 (1958) – Chamaecytisusbiflorussubsp.borysthenicus (Gruner) Elenevsky & Radygina in Elenevsky et al., Rast. Saratov. Pravober.: 41 (2000).

##### Type.

**Figure 4.**
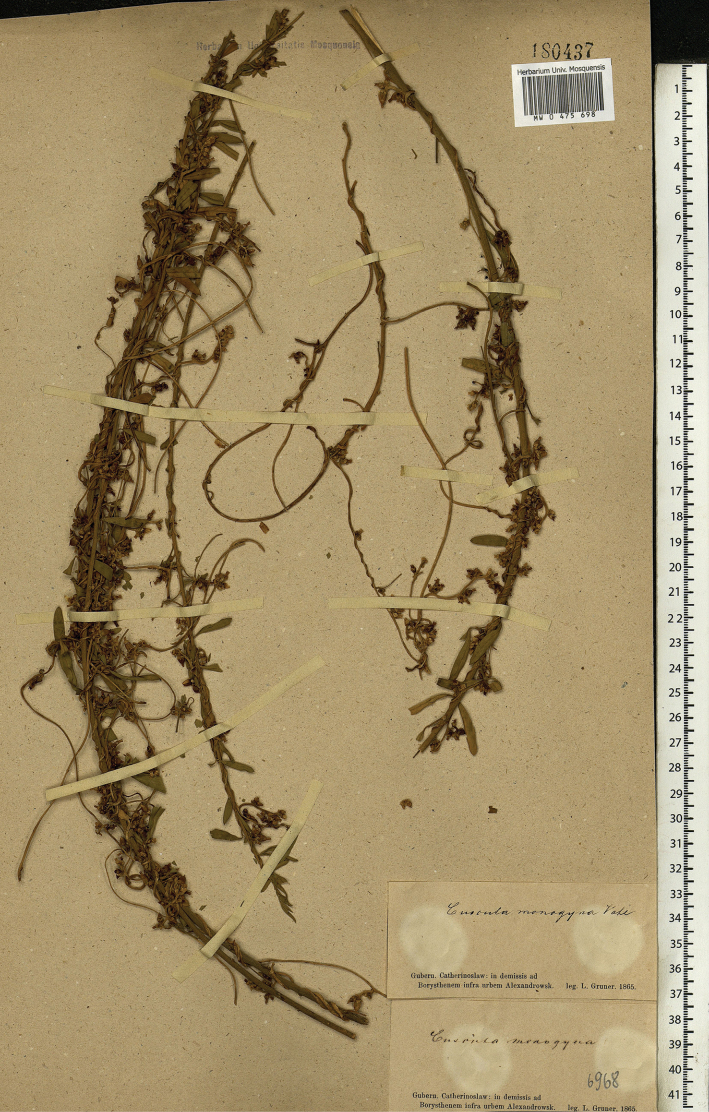
Lectotype of *Cytisusborysthenicus* Gruner.

Ukraine. Zaporozhie Region: “In demissis ad Borysthenem infra urbem Alexandrowsk [Zaporozhie],” [26.07].1865, *L. Gruner* (lectotype MW0475698, designated here). Fig. 4.

##### Description.

Upright shrubs with erect stems up to 120(200) cm tall and long branches. Leaves with lanceolate leaflets, densely and evenly hairy above, with dense appressed hairs 0.1–0.2(0.3) mm long below, petioles densely covered with appressed hairs. Flowers strictly lateral, 1–4 in axils, on pedicels 2–5 mm long, yellow; calyx 10–12 mm long, with appressed hairs 0.4–0.6 mm long; standard suborbicular, hairy above.

##### Distribution.

Europe: Ukraine, Crimea (Yena and Khlevnaya 2015a, 2015b; Fedoronchuk 2022), Russia (southern part: Kreczetowicz 1940; Borisova 1964). Asia: Russia (north-western Caucasus: Kreczetowicz 1940; Grossheim 1952; Ivanov 2019; south-western Siberia: Kreczetowicz 1940; Kurbatsky 1994), Kazakhstan (north-western part: Tulaganova 1981). New to Bashkiria, Bryansk and Kursk Regions of Russia. The presence in Belarus and European Kazakhstan is expected, but not confirmed.

##### Ecology.

Alluvial sands in larger river valleys, sandy steppes, open sands, sparse pine forests on sands, mostly along rivers.

##### Chromosome counts.

2n = 100 (Semerenko 1984, as *Chamaecytisus* sp.); material collected from native populations in Ukraine; vouchers at KW.

##### Notes on nomenclature.

Leopold Gruner (Lipschitz 1950; Leonov et al. 2014) explored the flora of steppic, sandy and calcareous areas near the confluence of the Konka River with the Dnepr River (now Zaporozhie Region, Ukraine).

Gruner (1869a, 1872) found *Cytisusborysthenicus* in a single place between the Konka River and Alexandrowsk Town (now Zaporozhie), rather frequent on small hills of partly open sands. While describing the new species, Gruner (1869a: 137) left it unnamed; in the second part of his synopsis (Gruner 1869b: 446), he mentioned in a note under *Cuscutamonogyna* that the latter species was collected on *Cytisusborysthenicus*. Since both papers were part of the same work and it was the only species of *Cytisus* recognised in the territory, the name of that species has been commonly accepted as validly published with a cryptic reference to the description via the title of the work (Art. 38.12 and 38.14, see also Ex. 19 under Art. 38.11).

Gruner (1869a, 1869b) visited the locality of *C.borysthenicus* twice, on 20 June and 26 July 1865. He collected sterile twigs and only one flowering branch with three flowers during his first visit and observed abundant plants of *Cuscutamonogyna* on these shrubs during the second visit.

Herbarium collections of Leopold Gruner are known at LE and MW (Lipschitz 1950). A minor part of his collections is placed at KW (formerly at CW: Leonov et al. (2014)). Some specimens are deposited at OXF (Clokie 1964), acquired as part of the collections of William Wilson Saunders (Druce 1897).

Kreczetowicz (1941) stated that the type of this species name is kept in Moscow, but it was not found anywhere including MW (Gubanov 2002). Lipsky (1899) recorded 237 specimens collected by Gruner in Ukraine and accessioned to the collections of the Imperial Botanical Garden in Saint-Petersburg (now part of the Komarov Botanical Institute, LE). This figure is much smaller than the number of taxa recorded by Gruner in his work, meaning that his collection acquired by LE was highly incomplete. We were also not able to trace any specimen collected by Gruner and labelled as *C.borysthenicus* in any Herbarium.

As a matter of surprise, one specimen representing Gruner’s collection of *Cuscutamonogyna*, with *Cytisusborysthenicus* as a host plant, has recently resurfaced at MW. This specimen was clearly associated by Gruner with the protologue of *C.borysthenicus* and is, therefore, part of the original material of the latter name. Although the fragment of *C.borysthenicus* on this specimen is a sterile branch densely covered by a parasite, it is perfectly adequate to identify the species and may serve as lectotype.

The original description of *C.borysthenicus* is ambiguous. The ecology (sandy hills) and the hairy standard indicate this species as currently understood, whereas the obovate-lanceolate leaves, glabrous above, clearly refer to *C.ruthenicus*. This discrepancy was neglected by Paczoski (1914) and Kreczetowicz (1940), who resurrected the name *C.borysthenicus* and applied it to the psammophilous species with narrowly lanceolate leaves, hairy above. *Cytisusborysthenicus*, *C.ruthenicus* and their hybrid co-occur in the *locus classicus* of the first species (Sennikov and Tikhomirov 2024a), and the original description of *C.borysthenicus* was apparently based on specimens of both species.

So far, the original material of *C.borysthenicus*, which is taxonomically referable to *C.ruthenicus*, has not been found. Gruner’s specimen of *Cuscutamonogyna* on *Cytisusborysthenicus* apparently belongs to the species as established by Paczoski (1914), Kreczetowicz (1940), Tzvelev (1987) etc. To fix this species name in its established interpretation, we designate the only available element of the original material as lectotype.

##### Notes on taxonomy and distribution.

This species is largely confined to the systems of southern East European rivers and was probably dispersed with sand deposits. Its distribution extends much further north-east and north-west than was indicated by Tzvelev (1987).

#### 4. Cytisus semerenkoanus

Taxon classificationPlantaeFabalesFabaceae

Sennikov & Val.N.Tikhom., sp. hybr. nov.

8C59A6BF-46AF-54F3-A469-3081F5BF820A

urn:lsid:ipni.org:names:77336839-1

##### Type.

**Figure 5.**
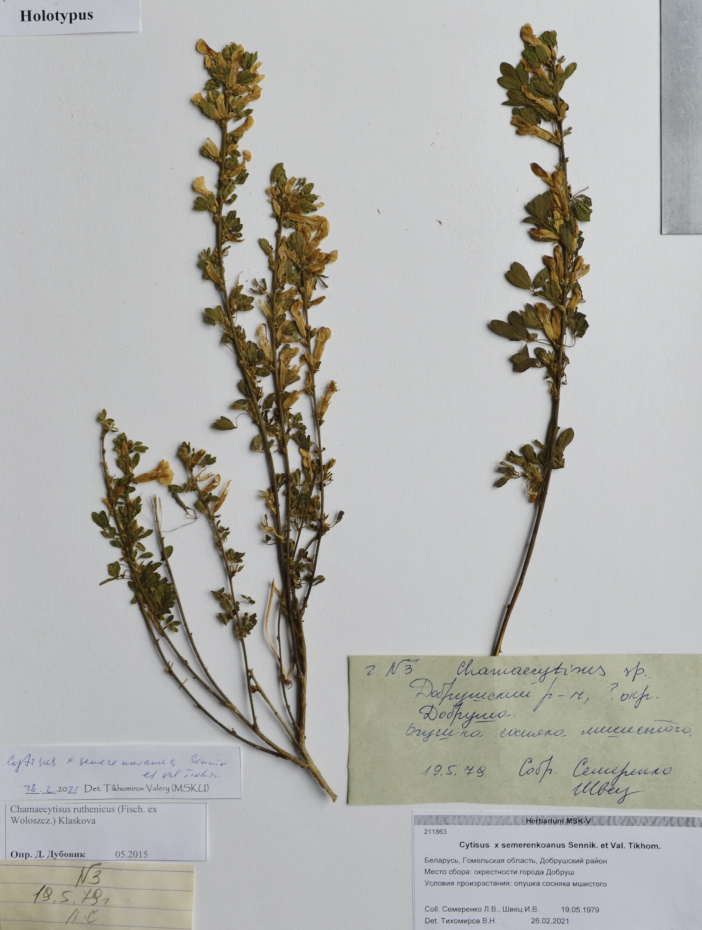
Holotype of *Cytisussemerenkoanus* Sennikov & Val.N.Tikhom.

Belarus. Gomel Region, Dobrush District, vicinities of Dobrush Town, margin of pine forest with moss cover, 19.05.1979, *L.V. Semerenko & I.V. Shvets* (holotype MSK, isotypes MSK, MSKU). Fig. 5.

##### Etymology.

The species name is given in honour of Larisa Vasilievna Semerenko (Parfionaŭ et al. 2018), who advanced our knowledge by her studies on the karyology and biology of *Cytisus* in Belarus.

##### Description.

Upright shrubs with erect, basally ascending stems up to 60(80) cm tall and long branches. Leaves with elliptic to obovate leaflets, sparsely hairy to subglabrous above, with lax hairs 0.2–0.6 mm long below, petioles sparsely covered with appressed and subpatent hairs. Flowers strictly lateral, 1–4 in axils, on pedicels 3–6 mm long, yellow; calyx 10–12 mm long, with appressed and subpatent hairs 0.4–0.9 mm long; standard suborbicular, hairy to subglabrous above.

##### Distribution.

Europe: Poland, Moldova, Belarus, Ukraine, Russia. Asia: Russia (Caucasus), Abkhazia.

##### Ecology.

In the forest zone, this taxon is found in dry forests on rich soils (oak forests and mixed broadleaved-pine forests with steppe plants), mostly in open places; in the forest steppe and steppe zones, it occurs in sparse forests and open steppe-like places.

##### Chromosome counts.

2n = 50 (Parfionaŭ et al. 1975, as *Chamaecytisus* sp.); material collected from native populations in Gomel Region; vouchers at MSK.

##### Notes on nomenclature.

Kreczetowicz (1940) described an alleged hybrid between *C.ruthenicus* and *C.elongatus* under the name *C.czerniaevii*, but the original material of the latter name belongs to *C.elongatus* rather than to the hybrid. For this reason, the hybrid is described here under a new name.

##### Notes on taxonomy and distribution.

Kreczetowicz (1940) described this taxon as a hybrid between *Cytisuslindemannii* (our synonym of *C.elongatus*) and *C.ruthenicus*, and this interpretation was accepted by Tzvelev (1987). Based on the intermediate morphology, we agree on the presence of hybrids between *C.ruthenicus* and *C.elongatus*. The distribution of *C.semerenkoanus* extends much further eastwards and northwards than the current distribution of its presumed parent, *C.elongatus*, which we explain by the extinction of the latter due to postglacial climate changes and hybridisation processes.

#### 5. Cytisus elongatus

Taxon classificationPlantaeFabalesFabaceae

Waldst. & Kit., Descr. Icon. Pl. Hung. 2: 200, t. 183 (1804)

40CFCCE9-B46E-5A53-941C-C3D7320235D4

**Figure 6.**
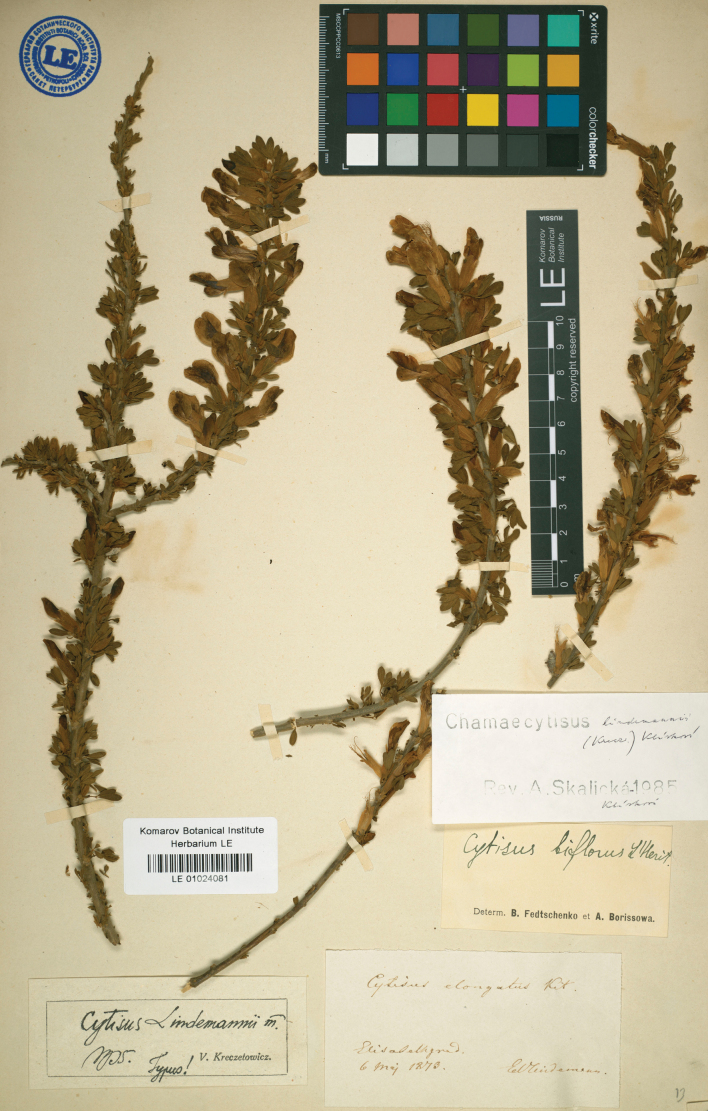
Holotype of *Cytisuslindemannii* V.I.Krecz.

– Chamaecytisus elongatus (Waldst. & Kit.) Link, Handbuch 2: 155 (1831) – Cytisushirsutussubsp.elongatus (Waldst. et Kit.) Briq., Etud. Cytis. Alp. Marit.: 168 (1894) – Chamaecytisusciliatussubsp.elongatus (Waldst. & Kit.) Soó in Feddes Repert. 85: 439 (1974) – Chamaecytisusglabervar.elongatus (Waldst. & Kit.) Kuzmanov in Jordanov, Fl. Narodna Republ. Bulg. 6: 86 (1976).
= Cytisus leucotrichus Schur in Oesterr. Bot. Z. 10: 179 (1860), syn. nov. – Chamaecytisusleucotrichus (Schur) Czerep., Sosud. Rast. SSSR: 229 (1981) – Chamaecytisustriflorussubsp.leucotrichus (Schur) Holub in Bertová, Fl. Slovenska 4(4): 35 (1988). Type. Romania. “Rothen Berg bei Mühlbach [Sebeș]”, [05].07.1853, *F. Schur* (lectotype LW00205768, designated by Pifkó (2009a: 153); isolectotype LW00205839).
= Cytisus lindemannii V.I.Krecz. in Bot. Zhurn. SSSR 25: 259 (1940), syn. nov. – Chamaecytisuslindemannii (V.I.Krecz.) Klásk. in Preslia 30: 214 (1958). Type. Ukraine. “Elisabethgrad” [Kropyvnytskyi], 06.05.1873, *E. Lindemann* (holotype LE01024081; isotype LE01024082). Fig. 6.
= Cytisus czerniaevii V.I.Krecz. in Bot. Zhurn. SSSR 25: 261 (1940), syn. nov. – Chamaecytisusczerniaevii (V.I.Krecz.) Tzvelev, Fl. Evropeiskoi Chasti SSSR 6: 223 (1987). Type. Ukraine. Kharkov Region, Zmiev District, Hamlet of Fedorchenko, 24.04.1910, *G.I. Širjaev* (lectotype KW000114840, designated here). Other original material. Ukraine. Kharkov Region: Steppes near Chuguev, 19.05.1852, *V.M. Cherniaev* (KW). Sumy Region, Lebedin District, “prope Grun, in steppis princ. Kapnist” [near Grun’, in steppes of Count Kapnist = ‘Mikhailovskaya Tselina’ Nature Reserve], 09.06.1905, *G.I. Širjaev* (KW000114839).
= Cytisus ponomarjovii Seredin in Novosti Sist. Vyssh. Rast. 13: 192 (1976), syn. nov. – Chamaecytisusponomarjovii (Seredin) Czerep., Sosud. Rast. SSSR: 229 (1981). Type. Russia. Krasnodar Territory, Tuapse District, 1 km NW of Dzhubga Village, oak forest, 08.07.1973, *R.M. Seredin* (holotype LE).
= Chamaecytisus korabensis Pifkó & Barina in Stud. Bot. Hung. 47(1): 164 (2016), syn. nov. Type. Albania. Qarku i Dibrës: [Korab-Koritnik Nature Park,] Mali i Bardhë Mts, near peak Maja e Pelpenikut, above village Sllatinë, on evaporites, 41.78419°N, 20.45978°E, 1928 m, 17.06.2013, *Z. Barina* & *D. Pifkó* 22354 (holotype BP759110; isotype BP759111).

##### Type.

Romania. Historical Banat Region: “In sylvis Beregh, Banaticis et Croaticis”, 1800, *P. Kitaibel* (lectotype W20030003241, left-hand fragment, designated here: https://w.jacq.org/W20030003241). Possibly Ukraine. [“In comitatis Bereghensis” = Bereg County, “in sylvis”,] Herb. Waldstein (superseded lectotype PR155757/738a, designated by Chrtek and Skočdopolová (1982: 226)).

##### Description.

Upright shrubs with erect, basally ascending stems up to 40–60(80) cm tall and long branches. Leaves with elliptic to obovate leaflets, densely hairy above, with lax hairs 0.4–0.8 mm long below, petioles rather densely covered with laxly appressed to subpatent hairs. Flowers strictly lateral, 1–4 in axils, on pedicels 2–4 mm long, yellow; calyx 11–12 mm long, with subpatent hairs 0.8–1.2 mm long; standard suborbicular, glabrous or hairy above.

##### Distribution.

Europe: France (along the valley of Rhône: Tison and de Foucault (2014)), Italy, Albania, Serbia, Greece, Bulgaria, Turkey (Cristofolini 1991), Bosnia and Herzegovina (new record), Montenegro (new record), Slovenia (new record), Croatia (Lovašen-Eberhardt 1997), North Macedonia (Micevski 2001), Austria (Cristofolini 1991), Hungary (Pifkó 2009b), Slovakia (Cristofolini 1991), Romania (Grinţescu 1957), Moldova (Heydemann 1986), Ukraine (Kreczetowicz 1940; Fedoronchuk 2022), Belarus (new record), Russia (south-western part) (Kreczetowicz 1940; Borisova 1964; Tzvelev 1987). Asia: Russia (western and central Caucasus: Grossheim (1952); Zernov (2006); Ivanov (2019)), Abkhazia (Kolakovsky 1985), Georgia (Ajaria: Gvinianidze (1981)), Turkey (Artvin Province: Kreczetowicz (1940)). Reported for the first time from Belarus and Bosnia and Herzegovina here. New to Bryansk and Lipetsk Regions of Russia. The actual distribution in Asian Turkey and the Balkans may be more extensive, but has been obscured due to the confusion with *C.hirsutus* (Gibbs 1970).

##### Ecology.

In the forest zone, this species occurs in sparse forest stands and on forest margins with steppe herbaceous species, mostly in xerophilous oak forests, at elevations below 500(700) m; in the forest steppe and steppe zones, it is found among sparse shrubs in dry creeks, steppe-like meadows and steppes. It also occurs in oak forests and steppe-like meadows in the mountains.

##### Chromosome counts.

2n = 50 (Semerenko 1984); material collected from native populations in Kursk and Lipetsk Regions; vouchers at MSK. Dubious record: 2n = 48 (Frahm-Leliveld (1957), as *Cytisuselongatus*); cultivated material; vouchers unknown.

##### Notes on nomenclature.

Skalická (1986) and Cristofolini (1991) accepted *Cytisustriflorus* Lam. as the priority name for this species. Its lectotype actually belongs to *C.hirsutus* L. (Sennikov and Tikhomirov 2024b).

*Cytisuselongatus* was described from present-day Romania (Caraş-Severin, Banat) and Ukraine (former Bereg County) (Waldstein and Kitaibel 1804). The original description of *C.elongatus* refers to plants with elongated branches and numerous flowers in lateral inflorescences, flowers shortly pedicellate and “slightly larger than in *C.supinus*”, branches with appressed hairs, leaves greyish-pubescent on both sides and calyces grey because of dense pubescence. The presumed original material (Pifkó 2007) is apparently heterogeneous, but the original description and drawing clearly indicate the intention to describe a species of *C.ratisbonensis* s.l. with the calyces having long subappressed pubescence and the leaves being hairy on the upper side, which unambiguously point at the species known as *C.lindemannii* (Tzvelev 1987) or *C.triflorus* (Cristofolini 1991).

According to the published diaries of P. Kitaibel (Gombocz 1945), he collected *C.elongatus* in Bereg County (7 July 1803, forest near Bereg, present-day Beregovo Town, mentioned as *C.elongatus*) and in Banat Region (26 July – 11 August 1800, many places, mentioned, according to Pifkó (2007), as *C.patens*). This means that the taxonomic concept of *C.elongatus* had been shaped on the basis of the Banat material prior to the travel to Bereg County. In Bereg County, besides the locality mentioned in the diary, where the plants were collected in fruits due to the late season, the species could have been collected anywhere on the route in northern and north-eastern Hungary.

After the protologue of *C.elongatus* was published, Kitaibel collected further specimens of this species (Lőkös 2001). In 1805, he travelled to Banat for the second time (5 July, near “Szlatina” = Slatina-Timiș, Caraș-Severin County, Romania, as *C.elongatus*). In 1815, he revisited Transcarpathia and collected in “Rhonaszegh” (6 August, Coștiui, Maramureș County, Romania, as *C.elongatus*) and near Bereg (25 September, Beregovo, Ukraine). The actual collections of Kitaibel may not have been limited to the localities mentioned in the diaries, but these data may be used as guidance to shape our understanding of the collections. For example, Kitaibel had an opportunity to collect the species during his three travels to the Matra Mts. and also in other travels that included present-day Croatia and Romania.

Chrtek and Skočdopolová (1982) designated a lectotype of *C.elongatus* from the collections of F. de Paula von Waldstein at the National Museum in Prague. The specimens kept as *C.elongatus* in this collection are accompanied by a generic label written by K. Sternberg, who possessed the collections after Waldstein’s death, whereas their original label data are lacking. Four plants are kept on two sheets under a single label. Of these plants, two were designated as a lectotype.

Chrtek and Skočdopolová (1982) preferred the designated sheet because the other one was a mixed collection of two different plants. However, they failed to observe that the two lectotype plants are also apparently different. The lanceolate leaflets of the right-hand plant of the lectotype are in apparent conflict with the protologue that states “foliolis obovatis”; besides, its inflorescence looks capitate rather than elongated as stated in the protologue (“totos ramos annotinos undique dense tegentes”). The other fragment agrees with the protologue in morphology, but there is no evidence that this particular material can be associated with the protologue and was not collected in any of the numerous later travels of Kitaibel. Due to the lack of the association with the protologue, the lectotype of *C.elongatus* designated by Chrtek and Skočdopolová (1982) cannot be accepted and should be superseded in favour of some certain element of the original material that is in agreement with the protologue.

In search for the other original material, we examined online collections of B, BP, PRC and W. Specimens in Herbarium Willdenow at B, which are labelled “Hungaria”, are likely original material because Willdenow received manuscripts and specimens from Waldstein and Kitaibel, of which hundreds are currently kept in Berlin (Hiepko 1972). Two of these specimens represent elongated branches, of which one (B-Willd 13622-03) has the leaves glabrous on the upper side and belongs to *C.cinereus*, whereas the other (B-Willd 13622-04) has the leaves hairy on the upper side and belongs to *C.triflorus* sensu Cristofolini. Plants collected from Bereg County are represented at PRC (PRC 454937), but their elongated branch has the leaves glabrous above and belongs to *C.cinereus*. Some original material collected in Banat is kept at BP (Pifkó 2007), including a specimen with elongated branches (Hb. Kitaibel XXIV: 161) collected near “Oravicza” (Oravița, Caraș-Severin County, Romania).

The most important specimen was found at W (W 20030003241). The plants on this sheet were identified as *C.elongatus* with a reference to the protologue; the label of this specimen written by Kitaibel is composite and reads “In sylvis Beregh, Banaticis et Croaticis”. This label reflects Kitaibel’s travels to Banat in 1800, to Croatia in 1802 and to Bereg County in 1803; it makes the specimen firmly linked to the protologue of *C.elongatus*. The sheet bears three fragments: a branch on the right side, densely leafy and abundantly flowering, corresponding to *C.cinereus*; a small fragment in immature fruit in the middle, also belonging to *C.cinereus* (possibly collected in 1803 from the locality in Bereg County mentioned in Gombocz (1945)); and an elongated branch in flower on the left side, whose calyces are villous and leaves are densely hairy above. The latter fragment fully agrees with the protologue of *C.elongatus*. We assume that the left-hand specimen belongs to the plants collected by Kitaibel in Banat in 1800 and used for the original description of *C.elongatus* and, therefore, designate it as a new lectotype.

This lectotype agrees with the usage in the Hungarian exsiccata (Kerner 1884; Anonymous 1919) and other specimens identified as *C.elongatus*, later usage favoured the application of this species name to *C.hirsutus* s.l. and the illustration was considered mismatching the original description (Kerner 1884). The usage of *C.elongatus* by Skalická (1986) and Pifkó (2009b) agrees with our lectotypification (except for their inclusion of plants belonging to *C.cinereus*); the placement of *C.elongatus* to the synonymy of “*C.triflorus*” by Cristofolini (1991) also agrees with our taxonomy.

The treatment of *C.leucotrichus* has been controversial. Schur (1859) described this plant as deviating from *C.hirsutus* by a denser “white” (sericeous) pubescence and smaller leaves. Tzvelev (1987) and Cristofolini (1991) placed it to the synonymy of *C.hirsutus*, in spite of its dense subpatent pubescence on branches and leaves (vs. sparse patent pubescence in *C.hirsutus*). Holub and Bertová (1988) accepted and subordinated it to *C.triflorus*, which was a name for *C.elongatus* at that time. The type collection of *C.leucotrichus* is represented by large branches in fruit, which are densely covered by subappressed (partly subpatent) hairs. This type of pubescence matches the characters of “*C.triflorus*” (Cristofolini 1991) and *C.lindemannii* (Tzvelev 1987) and agrees with the taxonomic concept of *C.elongatus* accepted here.

Although Kreczetowicz (1940) already employed the type concept, he did not indicate a type of *Cytisusczerniaevii*. Neither did he cite any specimen in the protologue; instead, he listed two localities in Kharkov Region. We found three specimens corresponding to those localities and identified by Kreczetowicz as *Cytisuslindemannii* × *C.ruthenicus*, in agreement with the hybrid origin of *C.czerniaevii* indicated in its protologue. One specimen was collected by Vasily Cherniaev and formerly deposited at CWU (which was transferred to KW after the Second World War), in the Ukrainian collections of Cherniaev which were extracted from his personal herbarium and placed within the main collections of KW for the preparation of *Flora of the Ukrainian SSR* (Krytzka et al. 2002). This specimen apparently provided the reason for naming the hybrid. Two specimens were collected by Grigory Širjaev in the former Kharkov Region of the Russian Empire (now Kharkov and Sumy Regions of Ukraine).

All the original material of *C.czerniaevii* belongs to *C.elongatus*. Kreczetowicz (1940) stated that his hybrid differed from the species by its subglabrous standard, which is, however, variable in *C.elongatus* (Wissjulina 1954). For this reason, the name *C.czerniaevii* cannot be used for a hybrid between *C.ruthenicus* and *C.elongatus*, but is a synonym of the latter.

Krytzka et al. (1999: 610) believed that the holotype of *C.czerniaevii* is kept at LE, but cited the species provenance from the protologue instead of the label data. Fedoronchuk et al. (2003) did not mention the presence of the original material of *C.czerniaevii* at KW. This material was recognised as such in 2012 by M. Shevera (on herbarium labels).

##### Notes on taxonomy and distribution.

Ledebour (1843) distinguished between the plants with appressed and subpatent hairs on the calyces, which he called *C.biflorus* L’Her. and *C.elongatus* Waldst. & Kit., respectively. The plants with the subpatent pubescence were reported from the steppe zone of Eastern Europe for the first time by Lindemann (1867), who used the nomenclature from Ledebour (1843).

Kreczetowicz (1940) believed that *C.elongatus* s. str. is replaced in steppes of Eastern Europe (Ukraine) and the North Caucasus by another taxon with a hairy (vs. glabrous) standard and a denser pubescence, which he named *C.lindemannii*. Skalická (1986) and Tzvelev (1987) accepted *C.lindemannii* in the same sense. Since this widely distributed species is variable in the length and density of pubescence and Kreczetowicz (1940) himself admitted that the pubescence on standard is variable within one species, we do not consider the western and eastern plants to be taxonomically different and restore the priority name for this species, *C.elongatus*. Cristofolini (1991) reduced *C.elongatus* to a synonym of “*C.triflorus*”, but placed *C.lindemannii* in the synonymy of *C.ruthenicus*; the latter decision is against the original description and type material of *C.lindemannii*, which has the subappressed to patent pubescence (vs. appressed in *C.ruthenicus*) and the leaves hairy above (vs. glabrous above in *C.ruthenicus*) (Kreczetowicz 1940).

Kreczetowicz (1940), Grossheim (1952) and Portenier and Solodko (2002) treated *C.hirsutissimus* as endemic to the Caucasus, a mountainous species which reportedly differed from the steppic, lowland East European *C.lindemannii* (= *C.elongatus*) in longer pedicels and a patent (vs. subappressed) pubescence of the whole plant. These minor and variable characters cannot be considered species-specific, and *C.hirsutissimus* of these authors was correctly identified with “*C.triflorus*” (= *C.elongatus*) (Cristofolini 1991).

Seredin (1976) described *C.ponomarjovii* as a local endemic of the western Caucasus and distinguished it from *C.caucasicus* by its denser pubescence. Cristofolini (1991) omitted this species, which was accepted in very few works (Czerepanov 1995; Ivanov 2019). Portenier and Solodko (2002) correctly noted that *C.ponomarjovii*, a species of lower elevations, corresponds to ‘*C.hirsutissimus* C.Koch’ of Russian authors (Kreczetowicz 1940; Grossheim 1952; Portenier and Solodko 2002), which is *C.triflorus* in the sense of Cristofolini (1991). We place it to the synonymy of *C.elongatus*, accordingly.

*Chamaecytisuskorabensis* was recently described by Pifkó and Barina (2016) as a local endemic of north-western Albania, which was considered as related to “the *C.ratisbonensis* and *C.triflorus* agg.” The protologue described and illustrated a minute plant collected at higher altitudes, with ascending stems covered by subappressed pubescence, leaves appressedly pubescent on both sides, and calyces 1–1.3 mm long with abundant subpatent hairs. These characters correspond to alpine forms of *C.elongatus*, which may be highly reduced in size in the subalpine mountain belt, whereas the differences in plant size played a major role in identification according to Pifkó and Barina (2016).

The earlier records of *C.lindemannii* from Belarus (Fedtschenko 1950) belong to *C.semerenkoanus*, but the presence of this species in the country is confirmed on the basis of recent collections.

##### Conservation status.

Although the species is not included in national or regional Red Lists, it occurs in some protected areas, for example, in the Mikhailovskaya Tselina Nature Reserve in Ukraine and in the Utrish Nature Reserve in Russia.

#### 6. Cytisus ratisbonensis

Taxon classificationPlantaeFabalesFabaceae

Schaeff., Bot. Exped.: tab. in prim. lib. (1760)

637F13AE-1403-58CB-9A90-CEAF7E3880F5

– Cytisus communis Lindem. in Bull. Soc. Imp. Naturalistes Moscou 40(1): 494 (1867), nom. illeg. superfl. – Cytisushirsutussubsp.ratisbonensis (Schaeff.) Briq., Étud. Cytises Alpes Mar.: 167 (1894) – Chamaecytisusratisbonensis (Schaeff.) Rothm. in Feddes Repert. 53(2): 143 (1944).

##### Type.

[icon] Schaeffer, Bot. Exped.: tab. in prim. lib. 1760 (presumably holotype).

##### Description.

Prostrate shrubs up to 20 cm above ground with long branches. Leaves with obovate to elliptic leaflets, glabrous above, with appressed hairs 0.4–0.8 mm long below, petioles densely covered with appressed hairs. Flowers strictly lateral, 1–4 in axils, on pedicels 3–5(7) mm long, pale yellow; calyx 11–14 mm long, with laxly appressed hairs 0.8–1.2(1.6) mm long; standard suborbicular, glabrous above.

##### Distribution.

Europe: Austria (Heywood and Frodin 1968), Germany (Heywood and Frodin 1968), Czech Republic (Skalická 1995; Kaplan et al. 2019), Hungary (Pifkó 2009b), Bulgaria (Heywood and Frodin 1968; Cristofolini 1991), Croatia (Lovašen-Eberhardt 1997), Romania (Cristofolini 1991), Slovakia (Holub and Bertová 1988), Poland (Heywood and Frodin 1968; Danielewicz 2020). Reported from Moldova (Heydemann 1986), but no specimens were seen by us from this country and its presence is considered unlikely. The records from the Balkans, Romania and Hungary include other related taxa and may be unreliable. The records from Belarus belong to *C.lithuanicus*. The records from Ukraine (Tzvelev 1987) belong to *C.lithuanicus* and *C.polonicus*. Most of the records from Poland Zieliński (1975) belong to *C.cinereus* and *C.polonicus*.

##### Ecology.

The species occurs in dry meadows among pine and oak mountain forests.

##### Chromosome counts.

2n = 48 (Dvořák and Dadákova 1976; Dvořák 1977); material from native populations collected in Czech Republic; vouchers at BRNU. The diploid counts (2n = 24) reported by Zieliński (1975) belong to *C.polonicus*. The tetraploid counts 2n = 48 reported by Zieliński (1975) belong to *C.cinereus*. The tetraploid counts 2n = 48 (Pogan et al. 1990), based on material from native populations collected in Poland, may belong to the same species (vouchers at KRAM, not controlled).

##### Notes on nomenclature.

The herbarium collections of Jacob Christian Schaeffer may be kept at REG. So far, the only, but unambiguous original element available to us is the illustration in the protologue.

##### Notes on taxonomy and distribution.

Before Kreczetowicz (1940), this species was treated very broadly to include many species of this group in Eastern Europe. Tzvelev (1987) and Semerenko (1999) still circumscribed this species too broadly, with the inclusion of *C.lithuanicus* which differs from *C.ratisbonensis* by its taller stems and shorter (up to 0.8 mm vs. 0.8–1.4 mm long) pubescence. Zieliński (1975) and Skalická (1995) treated *C.ratisbonensis* broadly, including plants with taller stems (up to 50 cm long) and larger flowers (calyx 10–13 mm long), which apparently belong to *C.lithuanicus* and *C.cinereus*. Holub and Bertová (1988) also included *C.ruthenicus* in this species. With exclusion of *C.polonicus*, *C.ratisbonensis* is treated as absent from Eastern Europe. It is retained in the present synopsis for the purposes of comparison.

#### 7. Cytisus polonicus

Taxon classificationPlantaeFabalesFabaceae

Sennikov & Val.N.Tikhom. sp. nov.

BCA412FF-AD99-55BB-BE1E-C530D1DCF568

urn:lsid:ipni.org:names:77336840-1

– Chamecytisus ratisbonensis auct.: Tzvelev 1989; Fedoronchuk 2022.

##### Type.

**Figure 7.**
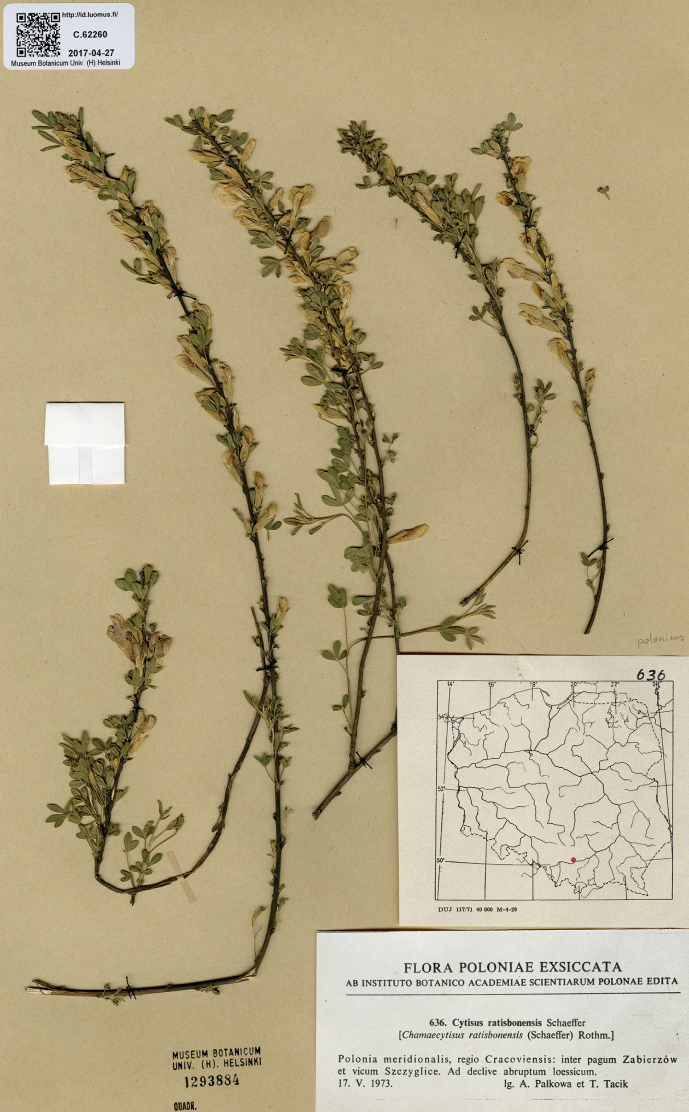
Holotype of *Cytisuspolonicus* Sennikov & Val.N.Tikhom.

Poland. “Regio Cracoviensis: inter pagum Zabierzów et vicum Szczyglice, ad declive abruptum loessicum, 17.05.1973, *A. Pałkowa & T. Tacik* [Flora Poloniae Exsiccata No. 636] (holotype H1293884; isolectotypes KRAM249040 and distributed to other herbaria). Fig. 7.

##### Etymology.

The new species is named after Poland, the country of its main distribution and type locality.

##### Description.

Prostrate shrubs up to 20 cm above ground with long branches. Leaves with obovate to elliptic leaflets, glabrous above, with appressed hairs 0.4–0.8 mm long below, petioles densely covered with appressed hairs. Flowers strictly lateral, 1–4 in axils, on pedicels 3–5(7) mm long, pale yellow; calyx (7)8–10 mm long, with (laxly) appressed hairs 0.6–0.8(1) mm long; standard suborbicular, glabrous above.

##### Distribution.

Europe: Poland, Ukraine. Its occurrence in western Belarus is expected due to the presence in Poland, 15 km from the border.

##### Ecology.

The species occurs in dry meadows or on calcareous denudations, on open slopes of hills and mountain foothills.

##### Chromosome counts.

2n = 24 (Zieliński (1975), as Cytisusratisbonensissubsp.ratisbonensis); material from native populations collected in Poland; vouchers at KOR and partly at KRAM.

##### Notes on taxonomy and distribution.

This species is most similar to *C.ratisbonensis*, from which it differs by its smaller flowers and shorter pubescence. It replaces the latter species in southern and eastern Poland and Ukraine.

#### 8. Cytisus cinereus

Taxon classificationPlantaeFabalesFabaceae

Host, Fl. Austriac.: 2: 343 (1831)

25ACA70B-E5E8-5D79-80A2-F19A4DA2918C

– Cytisus ratisbonensis subsp. cinereus (Host) Jáv., Magyar Fl. 2: 609 (1924).
= Cytisus horniflorus Borbás, Balaton Fl.: 299 (1900), syn. nov. Type. Hungary. “In arenosis silvaticis ad Monor in Hung. centrali”, 08.06.1887, *V. Borbás* (lectotype BP581457, designated by Pifkó (2005: 26)).
= Cytisus paczoskii V.I.Krecz. in Bot. Zhurn. SSSR 25: 261 (1940), syn. nov. – Chamaecytisuspaczoskii (V.I.Krecz.) Klásk. in Preslia 30(2): 214 (1958). Type. Ukraine. Ternopol Region: “Silva prope pag. Kidancy (non procul stat. viae ferrariae Maximovka)”, 26.04.1916, *A.I. Michelson* (holotype LE01024080).

##### Type.

**Figure 8.**
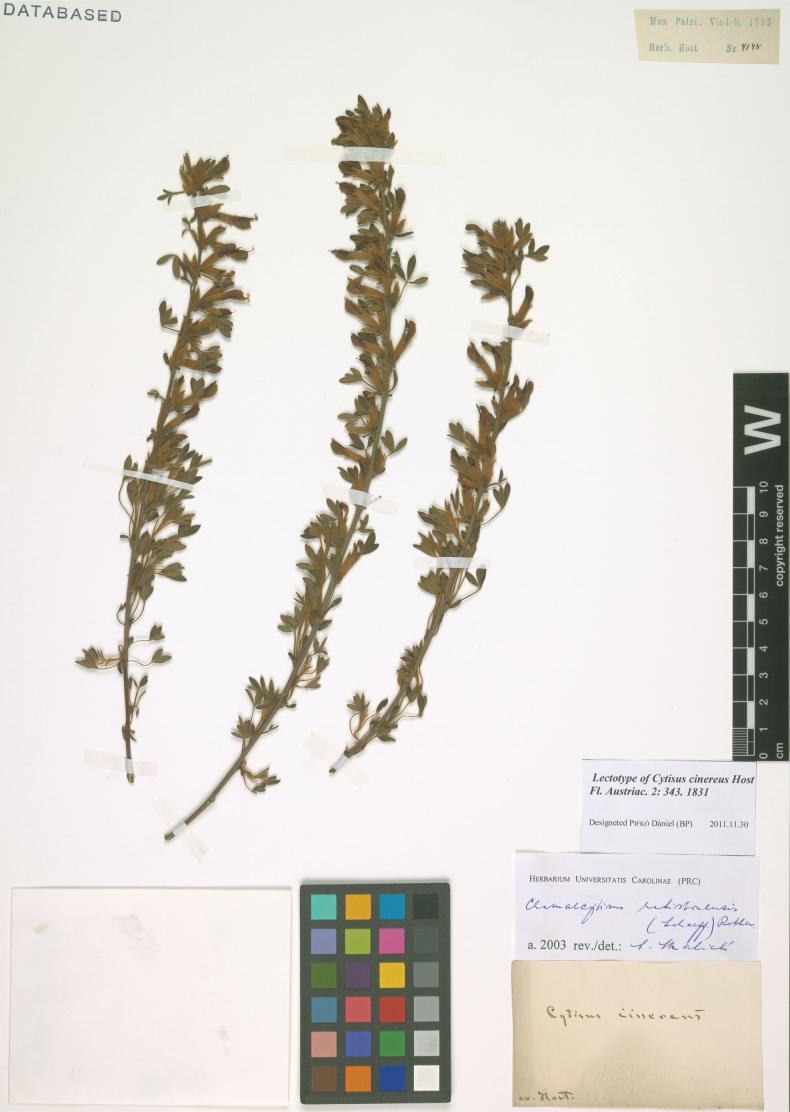
Lectotype of *Cytisuscinereus* Host.

Cultivation, originated from Hungary. “Ex Hort.” [Botanical Garden at Belvedere in Vienna, now Botanical Garden of the University of Vienna], Hb. Host 4148 (lectotype W1885-4148, designated here: https://w.jacq.org/W18850004148). Fig. 8.

##### Description.

Upright shrubs with erect, basally ascending stems up to 60(80) cm tall and long branches. Leaves with elliptic to obovate leaflets, glabrous above (the basal leaves are slightly hairy above), with appressed hairs 0.4–0.8(1.2) mm long below, petioles sparsely covered with laxly appressed hairs. Flowers strictly lateral, 1–4 in axils, on pedicels 3–5 mm long, yellow; calyx 11–14 mm long, with laxly appressed to subpatent hairs 0.6–1.2(1.5) mm long; standard subrbicular, glabrous or hairy above.

##### Distribution.

Europe: Austria, Slovakia, Serbia, Hungary, Romania, Poland, Ukraine (Tzvelev 1987; Fedoronchuk 2022), Moldova (Shabanova et al. 2014). As compared with the distribution area circumscribed by Tzvelev (1987), this species is new to Austria, Poland, Romania, Serbia, Slovakia and, due to the new synonymy, to Hungary. The only locality of this species previously reported from Moldova (Kreczetowicz 1940; Tzvelev 1987) is actually situated in Ukraine (Odessa Region); its voucher has not been found (Didukh 2009), but recent sources (Shabanova et al. 2014) reported a wide occurrence of the species in steppic areas of Moldova. The occurrence in Slovakia is logically expected.

##### Ecology.

The species occurs in open places, meadows and forest margins on plains and slopes of hilly uplands, often on sandy or calcareous substrates.

##### Chromosome counts.

2n = 48 (Zieliński (1975), as Cytisusratisbonensissubsp.ratisbonensis).

##### Notes on taxonomy and distribution.

*Cytisuscinereus* was described from sandy and forested areas of Hungary (Host 1831) with a reference to “*C.biflorus*” in Waldstein and Kitaibel (1804). The latter work lists a few localities in central and eastern Hungary, which are the likely origin of the material cultivated in Vienna by Host. Both descriptions (Waldstein and Kitaibel 1804: 181; Host 1831: 343) mentioned the oblong leaves glabrous above, a rather appressed pubescence on the calyx, and long erect branches. These characters agree with those of *C.paczoskii*; Kreczetowicz (1940) distinguished his latter species from *C.lindemannii* (= *C.elongatus*) on the basis of its glabrous standard (described as glabrous by Waldstein & Kitaibel, but stated as pubescent by Host).

*Cytisuscinereus* and *C.horniflorus* were distributed in the same exsiccatae as different taxa (Anonymous 1919), but the plants are virtually identical.

Kreczetowicz (1940) described this taxon as a presumed hybrid between *C.lindemannii* (= *C.elongatus*) and *C.ratisbonensis*. We consider it a stabile taxon with its own diagnostic characters and distribution area, clearly deserving the species rank. Some authors classified this species as an infraspecific taxon of *C.ratisbonensis* (Jávorka 1924) or included it in the latter species (Pifkó 2005, 2009b), from which it differs by erect branches, larger flowers on longer pedicels, and a longer and denser pubescence of the whole plant.

Skalická (1983) and Cristofolini (1991) correctly recognised *C.paczoskii* (= *C.cinereus*) as a species close to *C.ruthenicus*, but different in a more developed pubescence. Due to the lack of material, they were not able to circumscribe its distribution.

In Poland, Zieliński (1975) identified plants of this species as C.ratisbonensissubsp.ratisbonensis, and so did Pifkó (2005, 2009b) in Hungary. For this reason, *C.paczoskii* (= *C.cinereus*) was treated as endemic to Eastern Europe (Tzvelev 1987). According to our data, its distribution includes the Pannonian Basin and the territories from the Podolian to Lesser Polish uplands.

Dubovik (2016) reported *C.paczoskii* as occurring in western Belarus. This record is based on a different interpretation of this species name, which Dubovik considered to belong to a presumed hybrid between *C.ratisbonensis* and *C.ruthenicus*. The plants identified as *C.paczoskii* by Dubovik largely belong to *C.lithuanicus*.

#### 9. Cytisus lithuanicus

Taxon classificationPlantaeFabalesFabaceae

Gilib., Hist. Pl. Europe 2: 275 (1798)

767BD239-E8D1-51AA-8218-C0E24F4361B1

– Chamecytisus ratisbonensis auct.: Tzvelev (1987); Fedoronchuk (2022).

##### Type.

**Figure 9.**
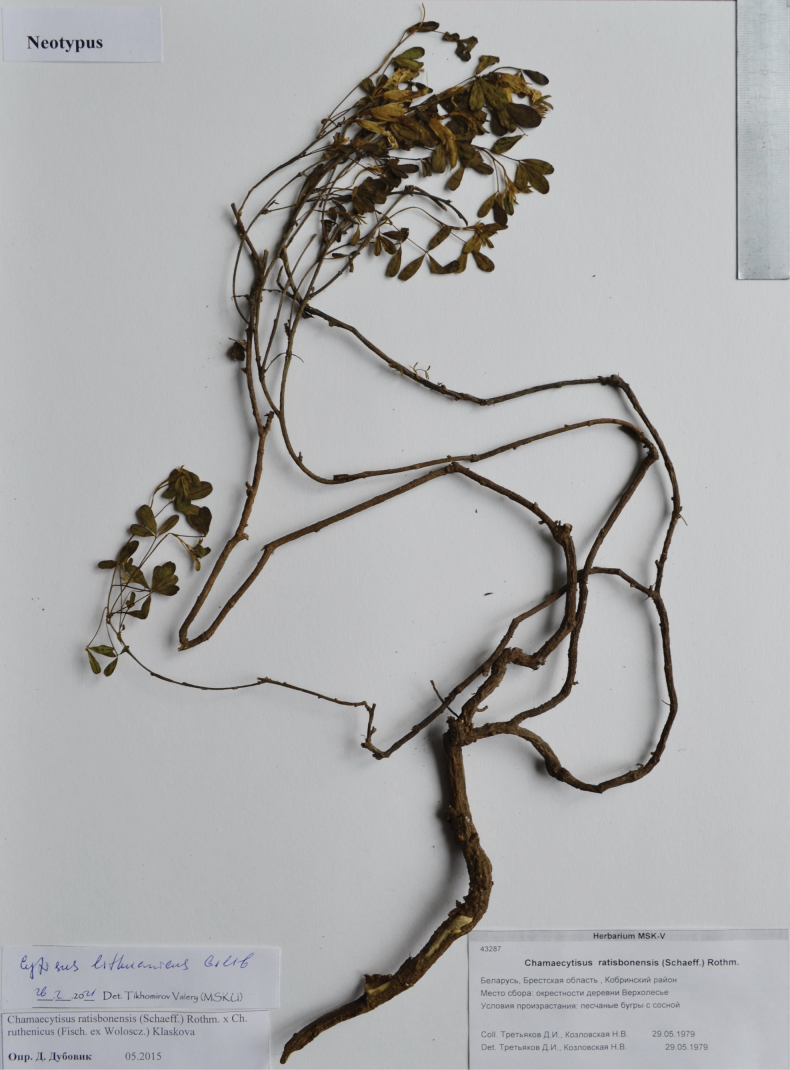
Neotype of *Cytisuslithuanicus* Gilib.

Belarus. Brest Region, Kobrin District. Vicinities of Verkholesie Village, sandy hills with pines, 29.05.1979, *D.I. Tretiakov & N.V. Kozlovskaya* (neotype MSK, designated here; isoneotypes MSK, MSKU). Fig. 9.

##### Description.

Upright shrubs with basally prostrate stems up to 40(60) cm tall and short branches. Leaves with obovate leaflets, glabrous above, with appressed hairs 0.4–0.6(0.8) mm long below, petioles sparsely to densely covered with laxly appressed hairs. Flowers strictly lateral, 1–4 in axils, on pedicels 5–10 mm long, pale yellow; calyx 12–14 mm long, with laxly appressed hairs 0.6–0.8 mm long; standard broadly elongate, glabrous above.

##### Distribution.

Europe: Poland, Belarus, Ukraine. This is the first attempt to circumscribe the distribution area of this species.

##### Ecology.

The species occurs in margins of dry pine and mixed forests.

##### Chromosome counts.

2n = 100 (Parfionaŭ et al. (1975), as *Chamaecytisus* sp.); material collected from native populations in Brest Region of Belarus; vouchers at MSK.

##### Notes on nomenclature.

The first name intended for this species, *Cytisuspubescens* Gilib., was originally introduced in Gilibert (1781), which is included in the list of suppressed works for species and infraspecific taxa, but validly published in a revised version of the same book (Gilibert 1793), which is not suppressed for nomenclatural purposes. Its intended replacement name, *C.lithuanicus*, was validly published in a generally accepted work of the same author (Gilibert 1798). Although the protologue of *C.lithuanicus* essentially recapitulated the information from the protologue of *C.pubescens*, it included no reference to the latter, whereas one of its elements, the illustration of *Cytisus VII* (Clusius 1601), was no longer considered taxonomically identical to the plants observed by Gilibert. As a result of these changes, *C.lithuanicus* is not a superfluous replacement of *C.pubescens*.

Gilibert (1781, 1798) provided an extensive morphological description of the species, which was poorly understood by subsequent authors because of the uncertain taxonomy of *Cytisus* in Belarus and Poland (Syreitschikow 1912; Kreczetowicz 1940). In eastern Poland and western Belarus, four species of C.sect.Tubocytisus may occur: octoploid (*C.lithuanicus* in our work), tetraploid (*C.cinereus* and *C.ruthenicus*) and diploid (*C.polonicus*) (Sennikov and Tikhomirov 2024a). To understand which of these four species was described by Gilibert, we compared the diagnostic characters extracted from the protologue of *C.lithuanicus* with the characters used as diagnostic in our work (Table 2).

**Table 2.** Comparisons of selected diagnostic characters from the protologue of *Cytisuslithuanicus* (Gilibert 1781, 1798), interpreted using Stearn (1983), with those of *C.lithuanicus*, *C.polonicus* and *C.ruthenicus* (this work).

Characters / species	*C.lithuanicus*, protologue	*C.cinereus*	*C.lithuanicus*, our work	*C.polonicus*	*C.ruthenicus*
Habit	“frutex basi decumbens sed rami erecti”	erect, basally ascending, not prostrate	basally prostrate, with erect branches	prostrate	erect
Plant height	“pedalis & cubitalis” = 30–45 cm	up to 40–60(80) cm	up to 40(60) cm	up to 20 cm	up to 120(200) cm
Calyx length	“sex linearum” = 13.5 mm	11–14 mm	12–14 mm	(7)8–10 mm	10–12 mm
Peduncle length	“vix quator linearum” = less than 9 mm	3–5 mm	5–10 mm	3–5(7) mm	5–7 mm

The habit of *C.lithuanicus* described in the protologue agrees with the octoploid species, whereas the match with *C.ruthenicus* (tall erect shrub) is impossible and the correspondence with *C.polonicus* (prostrate shrub) is less likely. The most important character is the calyx length, which immediately rejects *C.polonicus* (shortest calyces), but perfectly matches the octoploid (longest calyces). The peduncle length also disagrees with *C.polonicus*, which typically has shorter pedicels (subsessile flowers), whereas the octoploid plants usually have longer pedicels (lax flowers). *Cytisuscinereus* is similar to the plant described by Gilibert in the calyx length, but its stems are usually taller and pedicels are shorter; besides, the pubescence on the calyces of *C.cinereus* is long and laxly appressed to subpatent, and is usually perceived as golden-coloured because of its length and density (Kreczetowicz 1940; Heywood and Frodin 1968; Tzvelev 1987), whereas the calyces of *C.lithuanicus* were described as “albescens”, thus indicating a shorter and sparser pubescence like in the octoploid plants.

All these characters strongly indicate that the only species corresponding to the protologue of *C.lithuanicus* can be the octoploid, for which we resurrect this species name here.

*Cytisuslithuanicus* was described from the western vicinity of Białystok (present-day Poland), which was part of the Grand Duchy of Lithuania at the time of description. The original material was missing in the personal herbarium of Gilibert (KW) already by the beginning of the 20^th^ century (Syreitschikow 1912; Shiyan et al. 2013). In the absence of any material suitable for lectotypification, we designate as neotype a specimen matching the original description and belonging to the population which was caryologically tested.

##### Notes on taxonomy and distribution.

This species was formerly included in *C.ratisbonensis* (Kreczetowicz 1940; Zieliński 1975; Tzvelev 1987) because of its morphological similarity. *Cytisuslithuanicus* differs from *C.ratisbonensis* and *C.polonicus* by its upright stems, and also from the latter species by its longer calyces (12–14 vs. (7)8–10 mm long) and pedicels (5–10 vs. 3–5(7) mm long).

#### 10. Cytisus wulffii

Taxon classificationPlantaeFabalesFabaceae

V.I.Krecz. in Bot. Zhurn. SSSR 25: 262 (1940)

6396009B-6EB5-5832-8068-868B0766BE28

– Chamaecytisus wulffii (V.I.Krecz.) Klásk. in Preslia 30(2): 214 (1958).

##### Type.

**Figure 10.**
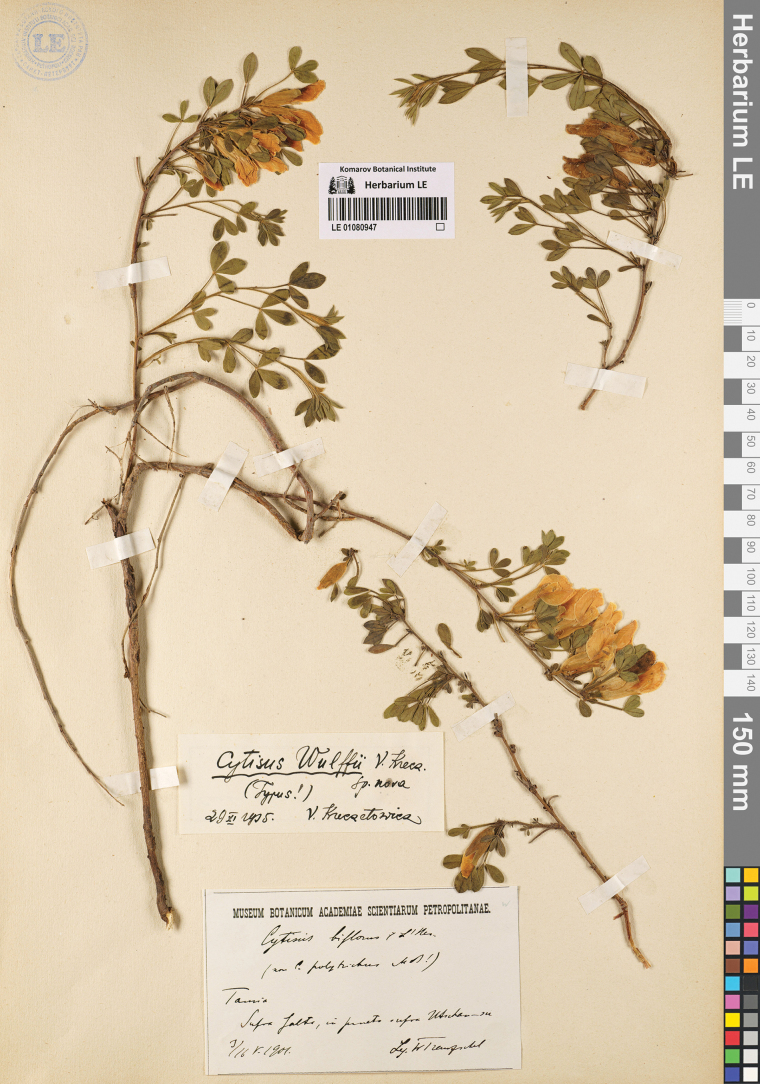
Lectotype of *Cytisuswulffii* V.I.Krecz.

Crimea. “Prope Yalta, in pineto supra Uchan-su”, 7/16.05.1901, *W. Tranzschel* (lectotype LE01080947, designated here; isolectotypes LE01080946, LE01080948). Fig. 10.

##### Description.

Small prostrate shrubs with abundantly branching stems up to 20 cm above ground. Leaves with obovate to oblong leaflets, hairy above, with numerous appressed hairs 0.3–0.7 mm long below, petioles sparsely covered with appressed to spreading hairs. Flowers strictly lateral, 1–4 in axils, on pedicels 3–5 mm long, yellow; calyx 14–15 mm long, with laxly appressed hairs 0.5–1 mm long; standard subrotund, partly hairy above.

##### Distribution.

Europe: Crimea (Tzvelev 1987; Yena 2012; Fedoronchuk 2022).

##### Ecology.

The species occurs on open gravelly and rocky slopes and in alpine meadows at the upper limit of pine forests.

##### Chromosome counts.

Unknown.

##### Notes on nomenclature.

Kreczetowicz (1940) indicated the type of *Cytisuswulffii* in the protologue. He wrote “Typus” on two specimens of the type gathering, which are, therefore, syntypes.

##### Notes on taxonomy and distribution.

This species is most similar to *Cytisuspolytrichus* M.Bieb., which occurs in the same area in the Crimea, but in the upper mountain zone and differs by patent (vs. appressed) hairs on its calyces and pedicels. Populations of both taxa may locally overlap (Pifkó and Barina 2016). *Cytisuswulffii* was originally reported also from the neighbouring area in the north-western Caucasus (Kreczetowicz 1940; Grossheim 1952; Zernov 2006); these records were rejected (Tzvelev 1987) and referred mostly to *C.elongatus*, which may look similar, but differs in subpatent pubescence.

### Identification key to East European species of Cytisussect.Tubocytisus (*C.ratisbonensis* group)

**Table:** 

1	Leaves glabrous above	**2**
–	Leaves variously hairy above	**6**
2	Pubescence completely appressed, sometimes plants are subglabrous to totally glabrous; calyces with appressed hairs 0.4–0.6 mm long; stems erect, up to 1(1.2) m tall	***Cytisusruthenicus***
–	Pubescence with subappressed to subpatent hairs over 0.6 mm long; stems prostrate or erect and basally ascending, not so tall	**3**
3	Flowers smaller; calyces (7)8–10 mm long, hairs 0.6–0.8(1) mm long	***Cytisuspolonicus***
–	Flowers larger; calyces 11–14 mm long, hairs 0.6–1(1.6) mm long	**4**
4	Calyces with laxly appressed hairs 0.6–0.8 mm long	***Cytisuslithuanicus***
–	Calyces with laxly appressed or subpatent hairs 0.6–1.2(1.6) mm long	**5**
5	Calyces with laxly appressed hairs 0.8–1.2(1.6) mm long; stems procumbent, up to 20 cm above ground	***Cytisusratisbonensis* (outside Eastern Europe)**
–	Calyces with laxly appressed to subpatent hairs 0.6–1.2(1.5) mm long; stems erect, basally ascending, up to 60 cm tall	***Cytisuscinereus***
6	Calyx 14–15 mm long; stems procumbent, up to 20 cm above ground	***Cytisuswulffii***
–	Calyx 10–12 mm long; stems erect or basally ascending, 30–150 cm tall	**7**
7	Calyces with appressed or laxly appressed hairs up to 0.6(0.8) mm long	**8**
–	Calyces with mostly subpatent hairs up to 1.2 mm long	**9**
8	Leaflets lanceolate or narrowly lanceolate, densely and evenly hairy above	***Cytisusborysthenicus***
–	Leaflets lanceolate to elliptic, sparsely hairy to subglabrous above	***Cytisuskreczetoviczii***
9	Leaves densely and evenly hairy above; calyx with subpatent hairs 0.8–1.2 mm long	***Cytisuselongatus***
–	Leaves sparsely hairy to subglabrous or nearly glabrous above; calyx with appressed and subpatent hairs 0.4–0.9 mm long	***Cytisussemerenkoanus***

## Conclusions

Our treatment is a further development of Cristofolini (1991), which improves the taxonomic and distributional data from Eastern Europe and neighbouring territories, based on much greater sampling of herbarium specimens and observations, and also on the comprehensive examination of type specimens. It is largely congruent with Tzvelev (1987), but avoids excessive taxonomic splitting.

This revision provides a taxonomic backbone for further studies in Cytisussect.Tubocytisus. Much further work is still required to establish chromosome counts for all its taxa and to uncover their evolutionary history. Distribution areas in the Balkans and some areas of Central Europe (Slovakia, Hungary) are unclear because of the lumping approach in local treatments and require complete revision. Recent hybridisation processes remain understudied.

The taxa of *C.ratisbonensis* group can be distinguished by differences in leaf shape and pubescence and in calyx size and pubescence; life form and habit provide important complementary information. These taxa also differ in their distribution areas and in their preference for elevation, substrate and vegetation type.

## Supplementary Material

XML Treatment for Cytisus ruthenicus

XML Treatment for Cytisus kreczetoviczii

XML Treatment for Cytisus borysthenicus

XML Treatment for Cytisus semerenkoanus

XML Treatment for Cytisus elongatus

XML Treatment for Cytisus ratisbonensis

XML Treatment for Cytisus polonicus

XML Treatment for Cytisus cinereus

XML Treatment for Cytisus lithuanicus

XML Treatment for Cytisus wulffii

## Additional information

### Author ORCIDs

Alexander N. Sennikov https://orcid.org/0000-0001-6664-7657

Valery N. Tikhomirov https://orcid.org/0000-0003-1822-0557

### Data availability

The dataset of distributional records collected for the present work was published through the Internet Archive (available online: https://archive.org/details/cytisus-ratisbonensis-dataset).
